# Integrating Canopy Dynamics and Grain Nutritional Profiles Reveals Genotypic Variation in Rye (*Secale cereale* L.)

**DOI:** 10.3390/plants15213291

**Published:** 2026-10-25

**Authors:** Ignacio Matamala, Manuel Chacón-Fuentes, Rodrigo Díaz, Juan Carlos García, Daniel Martínez-Cisterna, Marcelo Lizama, Leonardo Bardehle

**Affiliations:** 1 Programa de Doctorado en Ciencias Agroalimentarias y Medioambiente, Facultad de Ciencias Agropecuarias y Medioambiente, Universidad de La Frontera, Av. Francisco Salazar 01145, Casilla 54-D, Temuco 4811230, Chile; ignacio.matamala@ufrontera.cl; 2 Departamento de Producción Agropecuaria, Facultad de Ciencias Agropecuarias y Medioambiente, Universidad de La Frontera, Temuco 4811230, Chile; juan.garcia@ufrontera.cl; 3 Agriaquaculture Nutritional Genomic Center, CGNA, Las Heras 350, Temuco 4780000, Chile; 4 Drones Agrícolas, Alessandria 01591, Temuco 4810275, Chile; diazsaez@gmail.com; 5 Centro de Investigación Biotecnológica Aplicada al Medio Ambiente (CIBAMA), Universidad de La Frontera, Av. Francisco Salazar 01145, Casilla 54-D, Temuco 4811230, Chile; d.martinez11@ufromail.cl; 6 Departamento de Investigación e Innovación, Spin Off Universitaria Myconativa, Freire 4940000, Chile; marcelolizamavera@gmail.com

**Keywords:** rye, UAV, NDVI, canopy monitoring, grain nutritional quality, amino acids, vitamins, genotype characterization

## Abstract

Rye (*Secale cereale* L.) is a resilient cereal of growing importance for sustainable agriculture; however, integrative studies combining field-based canopy monitoring with detailed grain nutritional characterization remain limited. This study characterized four rye genotypes (Generator, Bar Power, L-INIA, and Lunator) by integrating unmanned aerial vehicle (UAV)-derived normalized difference vegetation index (NDVI) measurements with agronomic and grain nutritional assessments. Canopy development was monitored on three sampling dates during the growing season under two sowing dates, while plant architecture, grain nitrogen and protein concentrations, amino acid composition, and vitamin profiles were characterized at the genotype level. Significant genotypic differences were observed across multiple traits. Generator consistently exhibited high NDVI values and showed the highest grain nitrogen (2.74%) and estimated crude protein (approximately 15.96%) concentrations. L-INIA exhibited the greatest plant height (43.4 cm), largest leaf area (31.0 cm^2^), and highest total water-soluble vitamin concentration (223 mg 100 g^−1^ DW), whereas Bar Power accumulated the highest total fat-soluble vitamin concentration (81 µg g^−1^ DW). Lunator generally displayed intermediate values across the evaluated traits. Although total amino acid pools did not differ significantly among genotypes, individual amino acids showed distinct genotype-specific patterns. Collectively, these results demonstrate that temporal canopy development and grain nutritional composition capture complementary dimensions of genotypic variation in rye. Exploratory correlations between temporal NDVI descriptors and grain nutritional traits revealed no statistically significant associations after correction for multiple comparisons, indicating that canopy dynamics did not provide a validated predictor of grain nutritional composition in the evaluated genotypes. Under the environmental and management conditions evaluated, integrating temporal UAV-based canopy monitoring with nutritional profiling provided a comprehensive approach for characterizing genotype-specific agronomic and nutritional attributes relevant to rye improvement.

## 1. Introduction

Agricultural systems are increasingly challenged to produce food and feed with fewer external inputs while maintaining resilience under increasing climate variability [1,2]. This challenge has renewed interest in crop species capable of sustaining production under marginal or low-input conditions while providing grain with high nutritional value. Among temperate cereals, rye (*Secale cereale* L.) is particularly notable for its tolerance to low-fertility soils, drought-prone environments, cold conditions, and variable management regimes [3,4]. Historically cultivated for grain, forage, cover cropping, and soil protection, rye is increasingly recognized as a valuable component of sustainable agricultural systems, particularly in regions where environmental constraints can limit the performance of other cereals [5]. Globally, rye is the sixth most widely cultivated cereal crop, with a production of approximately 14.3 million tonnes in 2020 [6]. In addition to its agronomic resilience, rye grain is increasingly valued for its nutritional and functional properties [6]. It can provide relevant concentrations of protein, minerals, dietary fiber, vitamins, bioactive compounds, and nutritionally important amino acids for food and feed applications [7]. Its capacity to maintain productivity under low-input conditions and tolerate relatively poor soil fertility and water availability further supports its role in diversified production systems [6]. Rye is also widely used as livestock feed, particularly where environmental limitations constrain the cultivation of other cereals, making grain nutritional composition an important determinant of its feed value. Protein concentration, amino acid composition, and vitamin content can influence nutritional value, nutrient utilization, and animal performance [8,9]. Rye can also produce substantial above-ground biomass under low-input field conditions, further supporting its use as a forage resource and feedstock in integrated crop–livestock systems [9]. Consequently, identifying rye genotypes that combine favorable agronomic characteristics with differentiated grain nutritional profiles is increasingly relevant to breeding, animal nutrition, functional food development, and climate-smart agriculture [10,11]. However, agronomic and nutritional traits are influenced by the interaction among genotype, environment, and management. Protein concentration, amino acid composition, vitamin accumulation, biomass production, and related traits may vary with environmental and management conditions, including temperature, radiation availability, nutrient acquisition, and phenological development [12,13]. These relationships remain comparatively underexplored in rye relative to major cereals such as wheat, maize, and barley, for which agronomic and nutritional characterization has received considerably greater attention [14]. Rye research has traditionally emphasized traits such as yield potential, winter hardiness, forage production, and disease resistance, whereas studies simultaneously characterizing crop development and detailed grain nutritional composition remain comparatively limited [15,16,17,18]. This creates a need for approaches capable of capturing complementary dimensions of genotypic variation rather than evaluating agronomic or nutritional traits in isolation. Grain nutritional quality is conventionally characterized using direct laboratory-based methods, including nitrogen determination for protein estimation and chromatographic techniques for the quantification of amino acids and vitamins. These approaches provide quantitative and compound-specific information with high analytical specificity; however, they require sample collection and laboratory processing and are generally applied to harvested grain, limiting their ability to describe temporal variation in crop development. Thus, while direct biochemical analyses remain essential for determining grain nutritional composition, complementary non-destructive approaches can provide additional information on crop development throughout the growing season. Advances in crop phenotyping provide such opportunities by enabling temporal characterization of crop development. Conventional field evaluation of cereal genotypes commonly relies on destructive biomass sampling, manual canopy measurements, and assessments conducted at discrete stages or harvest [19]. Although these approaches provide essential agronomic information, they are labor-intensive and may inadequately capture temporal variation in canopy development. Unmanned aerial vehicle (UAV)-based remote sensing provides a rapid and non-destructive complement to conventional field measurements [20]. UAV platforms equipped with multispectral sensors enable repeated, high-resolution characterization of crop canopies under field conditions [21,22]. Among the available spectral indices, the normalized difference vegetation index (NDVI) is widely used as an indicator of canopy greenness and has been associated with biomass accumulation, chlorophyll status, photosynthetic activity, and plant nitrogen status. In cereal crops, NDVI has been extensively applied to biomass estimation, stress detection, crop monitoring, and yield forecasting [23,24]. Despite these advances, most UAV-based studies in cereals have primarily focused on canopy development, biomass, stress responses, or yield-related traits [24]. Considerably less attention has been directed toward integrating temporal canopy information with detailed grain nutritional characterization, particularly for biochemical components such as amino acids and vitamins. This distinction is important because canopy characteristics and final grain nutritional composition represent different, potentially complementary dimensions of genotypic variation. Consequently, characterization based exclusively on canopy spectral information may overlook genotypes with distinctive nutritional attributes, whereas grain analyses performed only at harvest provide little information about temporal differences in crop development. Integrating these approaches within the same experiment could therefore provide a more comprehensive characterization of rye genotypes. Crop establishment and subsequent canopy development can also be influenced by management conditions, including sowing time [25]. Even relatively small differences in sowing time may expose plants to variation in temperature, photoperiod progression, thermal accumulation, and radiation interception during establishment and development [26], potentially affecting canopy expansion and phenological progression [27]. In the present study, the two closely spaced sowing dates (27 and 31 March) were not intended to represent contrasting early- and late-sowing scenarios, but rather to account for short-term variation in crop establishment timing within the same growing environment. Their inclusion in the temporal NDVI analysis allowed the effect of this establishment-time variation to be distinguished from genotype- and sampling date-related variation in canopy development. Specifically, the study combines: (i) repeated UAV-derived NDVI measurements across crop development and two sowing dates; (ii) agronomic traits related to plant growth and architecture; (iii) grain nitrogen and protein concentrations; (iv) detailed amino acid composition; and (v) water- and fat-soluble vitamin profiles across four rye genotypes. We hypothesized that rye genotypes would exhibit distinct temporal canopy, agronomic, and grain nutritional characteristics under the evaluated conditions and that integrating these complementary measurements would provide a more comprehensive characterization of genotypic variation than either canopy or nutritional assessment alone. Accordingly, the objectives were to: (i) characterize temporal NDVI dynamics of four rye genotypes across two sowing dates; (ii) compare agronomic and grain nutritional traits among genotypes; (iii) characterize genotype-specific amino acid and vitamin profiles; and (iv) determine the extent to which integrated canopy and nutritional characterization can distinguish contrasting genotype-specific attributes under the environmental and management conditions evaluated.

## 2. Results

### 2.1. Temporal Canopy Dynamics

**Figure 1:**
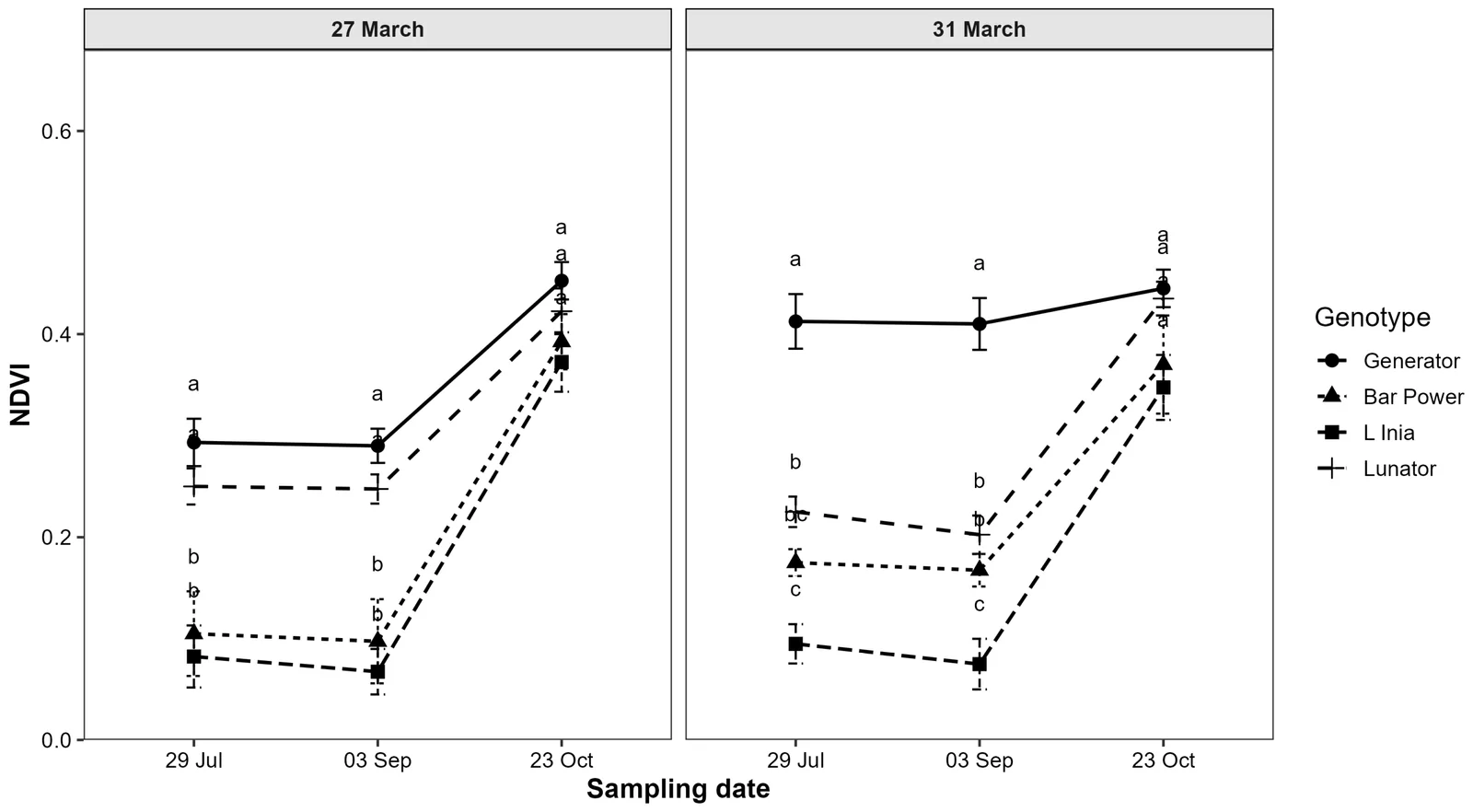
Temporal dynamics of UAV-derived normalized difference vegetation index (NDVI) in four rye genotypes under two sowing dates. Points represent means and error bars indicate standard error (SE). Different lowercase letters within each sampling date and sowing date indicate significant differences among genotypes according to Tukey’s test (*p* ≤ 0.05). Higher NDVI values indicate greater canopy greenness.

Four rye genotypes (Generator, Bar Power, L-INIA, and Lunator) were characterized through agronomic and grain nutritional assessments combined with temporal UAV-based canopy monitoring. Temporal canopy development was assessed under two sowing dates through three UAV-based multispectral flights conducted on 29 July, 3 September, and 23 October, providing temporal assessments of canopy development throughout the growing season. This integrative framework enabled the simultaneous characterization of temporal canopy dynamics and agronomic and grain nutritional traits. The mixed-effects analysis revealed significant effects of genotype (F = 33.92, *p* < 0.001) and sampling date (F = 470.56, *p* < 0.001) on NDVI, whereas the main effect of sowing date was not significant (F = 2.19, *p* = 0.151). Significant genotype × sampling date (F = 17.70, *p* < 0.001), sowing date × sampling date (F = 7.40, *p* = 0.0016), and genotype × sowing date × sampling date interactions (F = 4.08, *p* = 0.0022) indicated that temporal NDVI patterns differed among genotypes and varied partly according to sowing date. The genotype × sowing date interaction was not significant (F = 1.79, *p* = 0.176, Table S1). NDVI values changed markedly throughout the growing cycle and differed among genotypes (Figure 1). For both sowing dates, NDVI values were comparatively low during the first two evaluations (29 July and 3 September), followed by a clear increase at the final assessment (23 October), reflecting progressive canopy development toward spring conditions. Under the 27 March sowing date, Generator and Lunator showed the highest statistical grouping during the July and September evaluations. Generator increased from approximately 0.30 in July to 0.45 in October, while Lunator increased from 0.25 to 0.42. In contrast, Bar Power and L-INIA exhibited significantly lower early-season NDVI values, but both increased substantially by October, reaching approximately 0.39 and 0.37, respectively. A similar temporal pattern was observed under the 31 March sowing date. Generator showed the highest NDVI values during July and September, with values of approximately 0.41 at both evaluations, and reached approximately 0.45 in October. Lunator showed intermediate early-season values, decreasing slightly from approximately 0.23 in July to 0.20 in September before increasing to approximately 0.44 in October. Bar Power and L-INIA exhibited lower early-season values, approximately 0.18 and 0.10 in July and 0.17 and 0.08 in September, respectively, before increasing to approximately 0.37 and 0.35 in October. By the final October evaluation, no significant differences among genotypes were detected under either sowing date, indicating convergence in canopy greenness among the evaluated materials at the final sampling date. Overall, these results demonstrate genotype-dependent differences in temporal canopy development, with greater differentiation during earlier and intermediate evaluations and increasing convergence toward the final assessment (Figures S1 and S2). Additional temporal NDVI descriptors were calculated specifically for the 27 March sowing to characterize genotype-level canopy dynamics in relation to grain nutritional composition (Table S2). Generator exhibited the highest integrated NDVI (AUC = 29.06 NDVI·day), followed by Lunator (25.71), Bar Power (15.90), and L-INIA (13.70). The corresponding normalized AUC values were 0.338, 0.299, 0.185, and 0.159, respectively. All genotypes showed an increase in mean NDVI between July and October, with the largest changes observed in L-INIA (ΔNDVI = 0.290) and Bar Power (0.288), compared with Generator (0.159) and Lunator (0.173). These results demonstrate genotype-dependent differences in both cumulative canopy greenness and the magnitude of temporal NDVI changes.

### 2.2. Agronomic Traits at the Vegetative Stage

**Figure 2:**
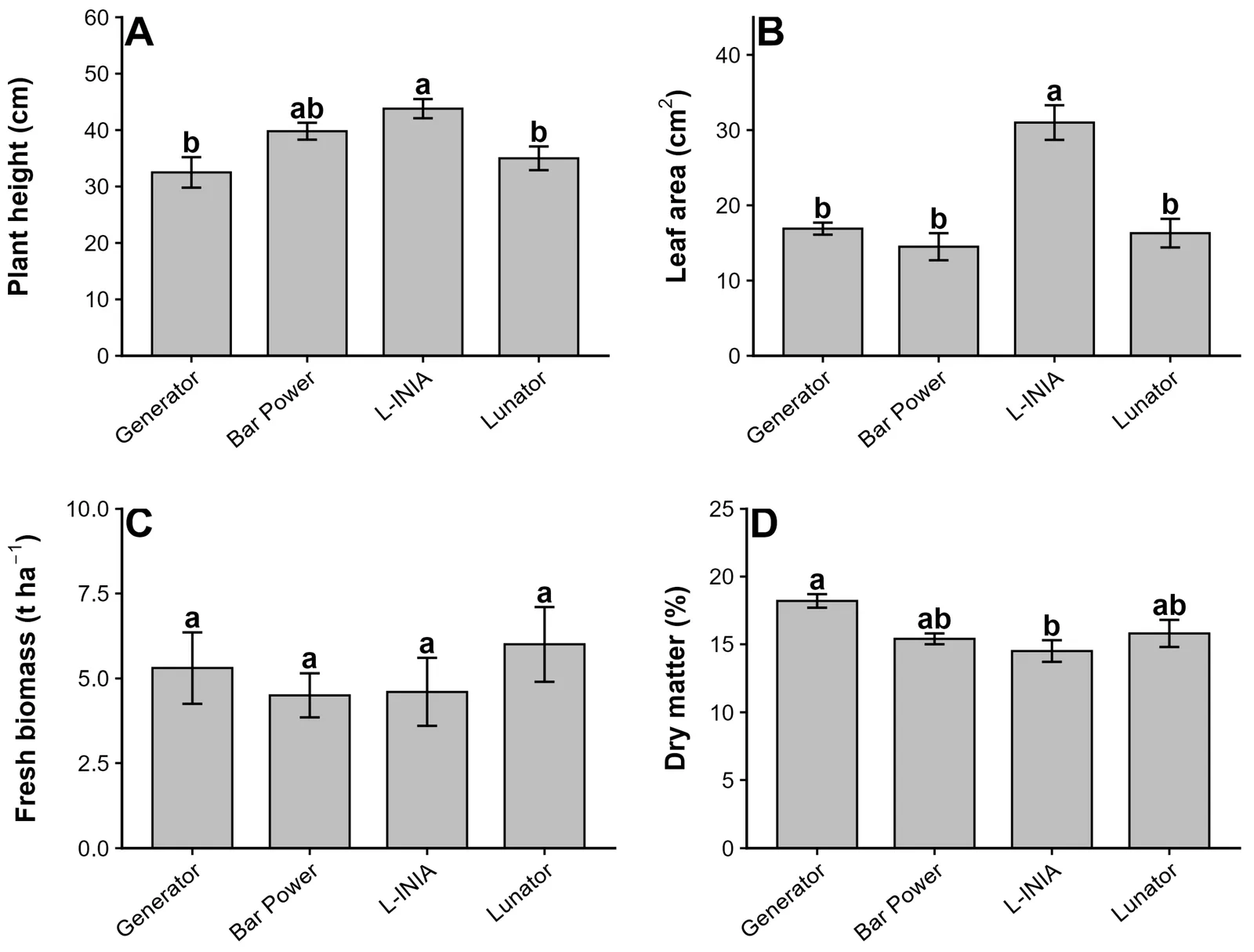
Genotypic variation in agronomic and biomass traits of rye evaluated under field conditions. Mean values of (**A**) plant height, (**B**) leaf area, (**C**) fresh biomass, and (**D**) dry matter are shown. Bars represent means and error bars indicate standard error (SE). Different lowercase letters above bars indicate significant differences among genotypes according to Tukey’s test (*p* ≤ 0.05). Plant height is expressed in cm, leaf area in cm^2^, fresh biomass in t ha^−1^, and dry matter as percentage (%).

Significant differences among genotypes were detected for plant height and leaf area, whereas fresh biomass did not differ significantly and dry matter showed moderate variation among genotypes (Figure 2). These measurements were obtained during the vegetative assessment conducted in the first week of August 2025 (BBCH 29–31). The observed differences in plant height reflect genotype-specific variation around the approximate 40 cm canopy height used as a general reference for scheduling the sampling. L-INIA was the tallest genotype and differed significantly from Generator and Lunator, whereas Bar Power showed an intermediate value (39.8 cm). Leaf area also varied markedly among genotypes. L-INIA presented the largest mean leaf area (31.0 cm^2^), nearly doubling the values observed in Generator (16.8 cm^2^), Lunator (16.2 cm^2^), and Bar Power (14.4 cm^2^). The latter three genotypes did not differ significantly from one another. Fresh biomass ranged from approximately 4.5 to 6.0 t ha^−1^, with no significant differences among genotypes (Figure 2). Generator exhibited the highest dry matter concentration (18.3%), whereas L-INIA showed the lowest value (14.5%), with a significant difference between these genotypes. Bar Power and Lunator showed intermediate values. Collectively, these results indicate clear genotype-dependent variation in plant architecture and dry matter accumulation, with L-INIA characterized by greater plant height and leaf area and Generator by a higher dry matter concentration.

### 2.3. Grain Nitrogen and Crude Protein Concentration

**Figure 3:**
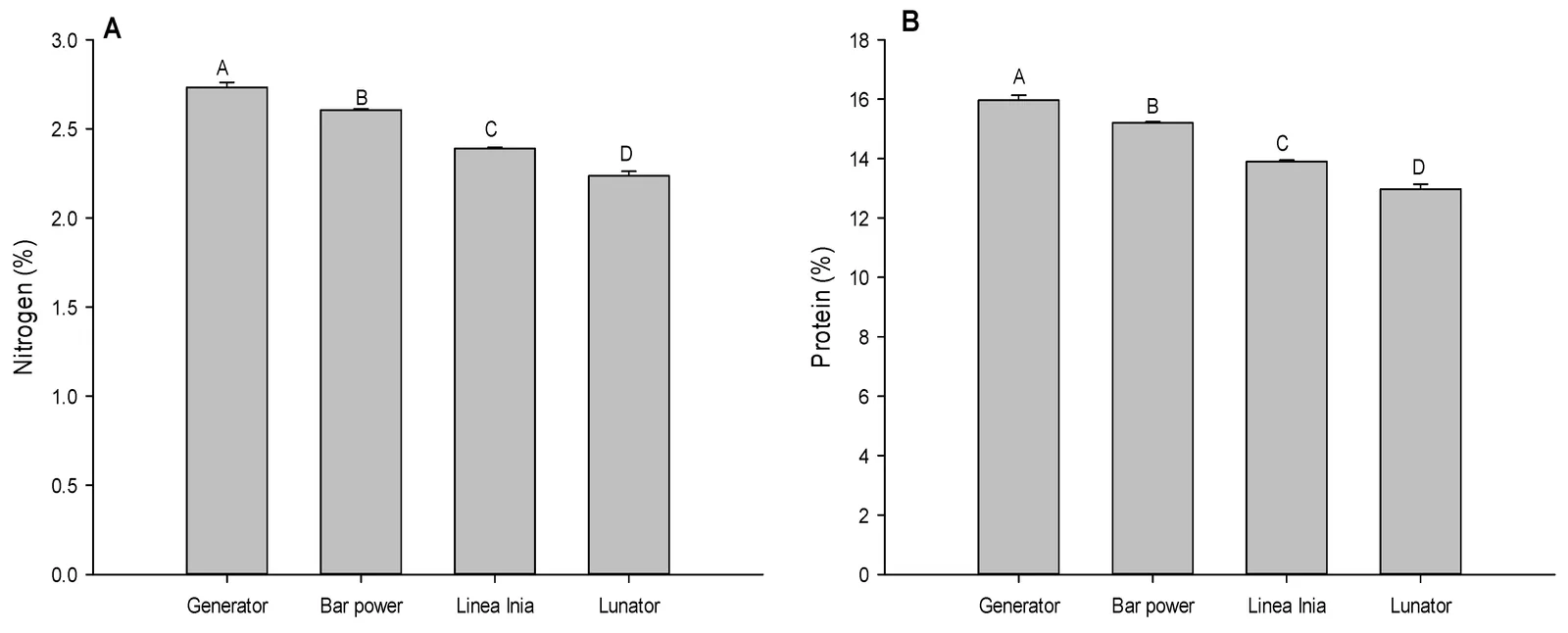
Grain nitrogen and crude protein concentrations of four rye genotypes. Mean values of (**A**) grain nitrogen concentration and (**B**) crude protein concentration are shown. Bars represent means and error bars indicate standard error (SE). Different uppercase letters above bars indicate significant differences among genotypes according to Tukey’s test (*p* ≤ 0.05). Values are expressed as percentages of dry weight (% DW).

Significant genotypic differences were observed for grain nitrogen and crude protein concentrations (Figure 3). Grain nitrogen concentration ranged from 2.24% in Lunator to 2.74% in Generator, with significant differences among genotypes. The same ranking was observed for crude protein concentration, reflecting its calculation from grain nitrogen values. Generator showed the highest estimated crude protein concentration (approximately 15.96%), followed by Bar Power (15.20%), L-INIA (13.90%), and Lunator (12.97%), based on the rye-specific nitrogen-to-protein conversion factor of 5.83. Thus, under the environmental and management conditions evaluated, Generator exhibited the highest grain nitrogen and crude protein concentrations, whereas Lunator showed the lowest values.

### 2.4. Amino Acid Composition

**Figure 4:**
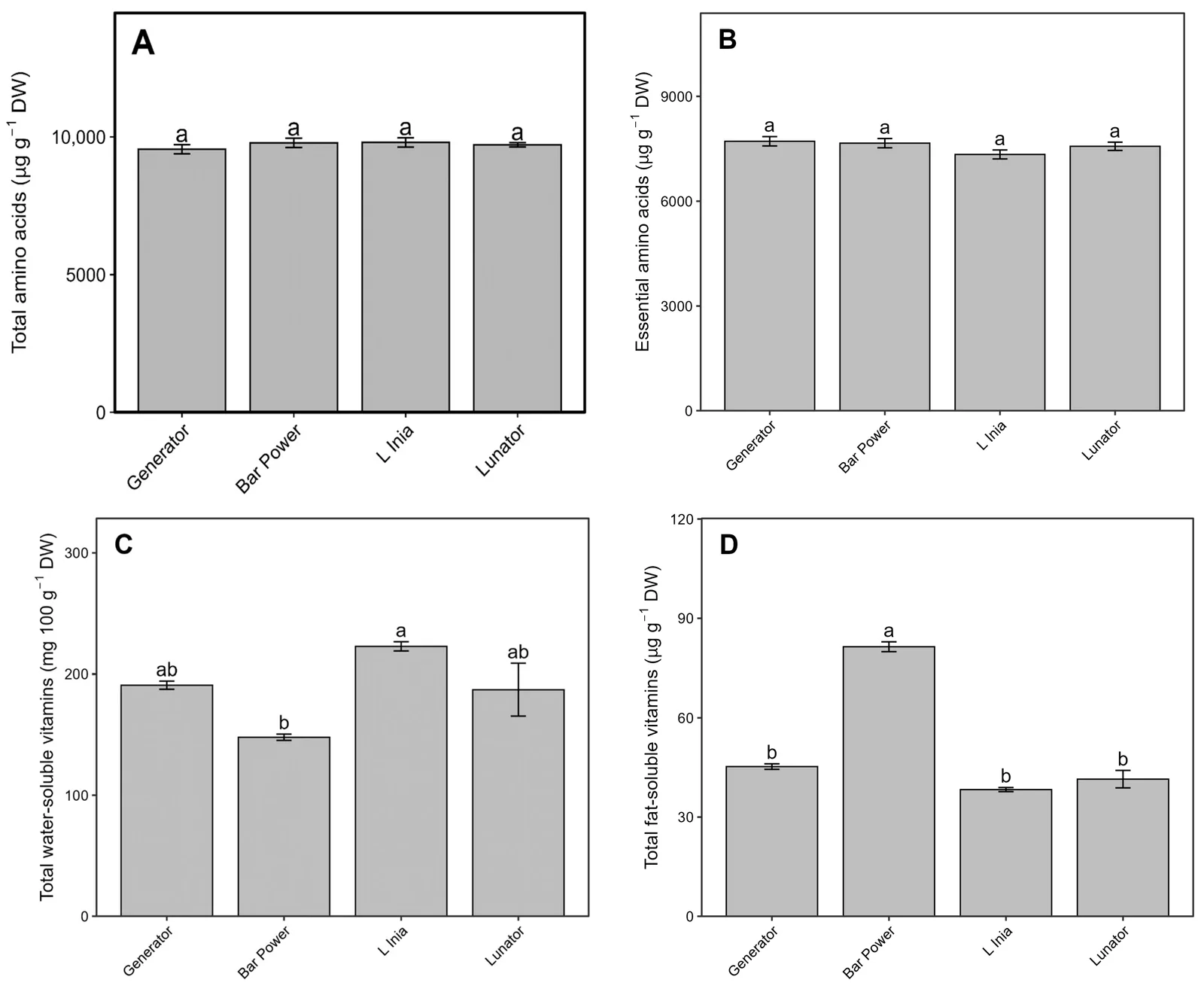
Free amino acid pools and vitamin composition of four rye genotypes. Mean values of (**A**) total free amino acids, (**B**) total essential free amino acids, (**C**) total water-soluble vitamins, and (**D**) total fat-soluble vitamin concentration refer exclusively to the combined concentrations of α-tocotrienol and vitamin K1, the two fat-soluble compounds quantified in this study. It does not represent the complete vitamin E profile or the total content of all fat-soluble vitamins. Bars represent means and error bars indicate standard error (SE). Different lowercase letters above bars indicate significant differences among genotypes according to Tukey’s honestly significant difference (HSD) test (*p* ≤ 0.05). Total free amino acids and total essential free amino acids are expressed as µg g^−1^ dry weight (DW), water-soluble vitamins as mg 100 g^−1^ DW, and fat-soluble vitamins as µg g^−1^ DW.

Despite differences in crude protein concentration, total free amino acid concentration did not differ significantly among genotypes (Figure 4A), remaining within a narrow range of approximately 9500–9800 µg g^−1^ DW. Similarly, total essential amino acid concentration did not differ significantly among genotypes (Figure 4B), ranging from approximately 7300 to 7700 µg g^−1^ DW. In contrast, analysis of individual amino acids revealed distinct genotype-specific compositional patterns (Table 1 and Table S3). Generator exhibited comparatively high concentrations of glutamine, glutamate, threonine, methionine, leucine, cysteine, and histidine. However, for glutamate and leucine, significant pairwise differences were detected only between Generator and Bar Power and between Generator and L-INIA, respectively. Bar Power showed comparatively high concentrations of arginine, serine, proline, and valine. L-INIA exhibited comparatively high concentrations of aspartate, glycine, alanine, isoleucine, and tyrosine, although its tyrosine concentration did not differ significantly from that of Bar Power (Table 1). Lunator generally displayed intermediate concentrations across individual amino acids. Lysine did not differ significantly among genotypes. Thus, although total free and essential amino acid concentrations were similar among genotypes, significant genotype-specific differences were detected in the concentrations of several individual amino acids. After applying the Benjamini–Hochberg correction for multiple testing, significant genotype effects were retained for 16 of the 18 individual free amino acids (FDR-adjusted *p* < 0.05), whereas phenylalanine and lysine remained non-significant (Table S3).

### 2.5. Vitamin Composition

Significant differences among genotypes were observed for total water-soluble vitamin concentration (Figure 4C). L-INIA showed the highest concentration (approximately 223 mg 100 g^−1^ DW), significantly exceeding Bar Power (149 mg 100 g^−1^ DW), whereas Generator and Lunator displayed intermediate values (191 and 188 mg 100 g^−1^ DW, respectively). Total fat-soluble vitamin concentration also differed significantly among genotypes (Figure 4D). Bar Power exhibited the highest combined concentration of the quantified fat-soluble compounds (81 µg g^−1^ DW), nearly twice the values observed in Generator, L-INIA, and Lunator (38–46 µg g^−1^ DW). These values represent the sum of α-tocotrienol and vitamin K1 and should not be interpreted as total fat-soluble vitamin content. Analysis of individual vitamins further revealed distinct genotype-specific compositional patterns (Table 2). L-INIA showed higher concentrations of vitamins C and B9, whereas Bar Power showed higher concentrations of vitamins B1 and B6 and α-tocotrienol. Generator was characterized by higher concentrations of vitamins B2 and K1, whereas Lunator generally displayed intermediate concentrations across the evaluated vitamins. Overall, these results demonstrate significant genotype-specific differences in both total vitamin concentrations and the composition of individual vitamins. All seven evaluated vitamins retained statistically significant genotype effects after correction for multiple testing using the Benjamini–Hochberg procedure (FDR-adjusted *p* < 0.05; Table S3).

### 2.6. Exploratory Associations Between Temporal NDVI and Grain Nutritional Composition

**Figure 5:**
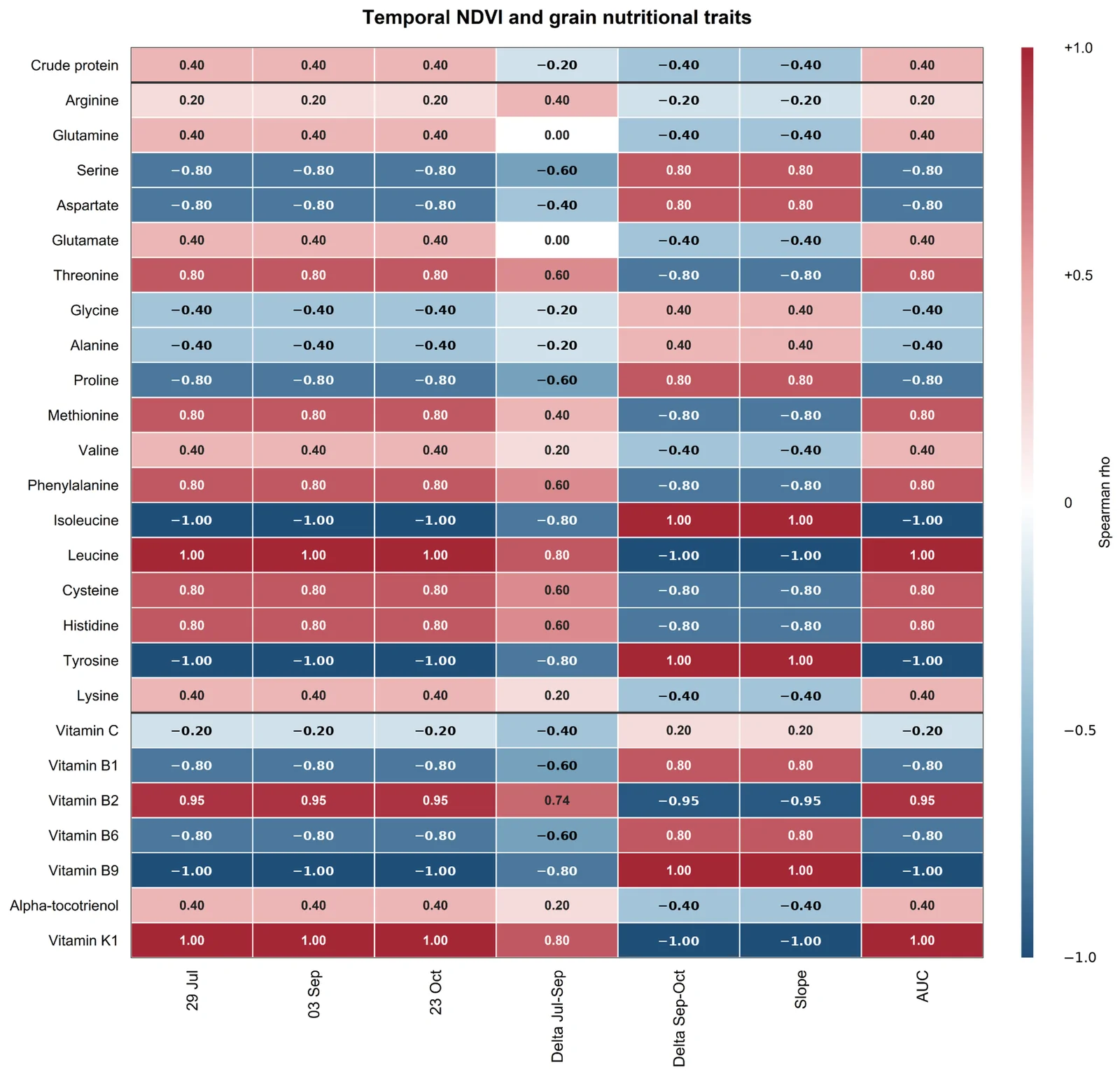
Exploratory associations between temporal canopy NDVI descriptors and grain nutritional composition in four rye genotypes. Heatmap of Spearman’s rank correlation coefficients (ρ) between UAV-derived NDVI at three acquisition dates, temporal NDVI changes, overall temporal slope, and area under the NDVI curve (AUC), and grain nutritional traits comprising crude protein, 18 individual free amino acids, and seven vitamins. Correlations were calculated using genotype-level means (*n* = 4) from the 27 March sowing. Red and blue indicate positive and negative associations, respectively. Exact two-sided permutation *p*-values were adjusted using the Benjamini–Hochberg procedure across 234 exploratory comparisons. No associations were statistically significant after correction (adjusted *p* ≥ 0.4875). These results do not represent paired plot-level relationships or evidence of predictive performance.

Exploratory Spearman rank correlation analyses were conducted between temporal NDVI descriptors from the 27 March sowing and grain nutritional traits, including crude protein, 18 individual free amino acids, and seven vitamins (Figure 5; Table S4). A total of 234 associations were evaluated using exact two-sided permutation tests. None reached statistical significance, either before or after Benjamini–Hochberg correction (minimum exact *p* = 0.0833; minimum adjusted *p* = 0.4875). The correlation heatmap (Figure 5) revealed several strong descriptive rank associations. Isoleucine, tyrosine, and vitamin B9 were negatively correlated with NDVI at all three acquisition dates and with AUC (ρ = −1.00), whereas leucine and vitamin K1 showed positive correlations (ρ = 1.00). Vitamin B2 also exhibited a strong positive association (ρ = 0.949). In contrast, crude protein and α-tocotrienol exhibited weaker associations with NDVI and AUC (ρ = 0.40). Nevertheless, none of these associations was statistically significant. As the analysis included only four genotype-level observations and did not rely on verified plot-level matching, the results describe exploratory covariation among genotype means rather than plot-specific or predictive relationships.

### 2.7. Integrated Multivariate Characterization of Genotypes

**Figure 6:**
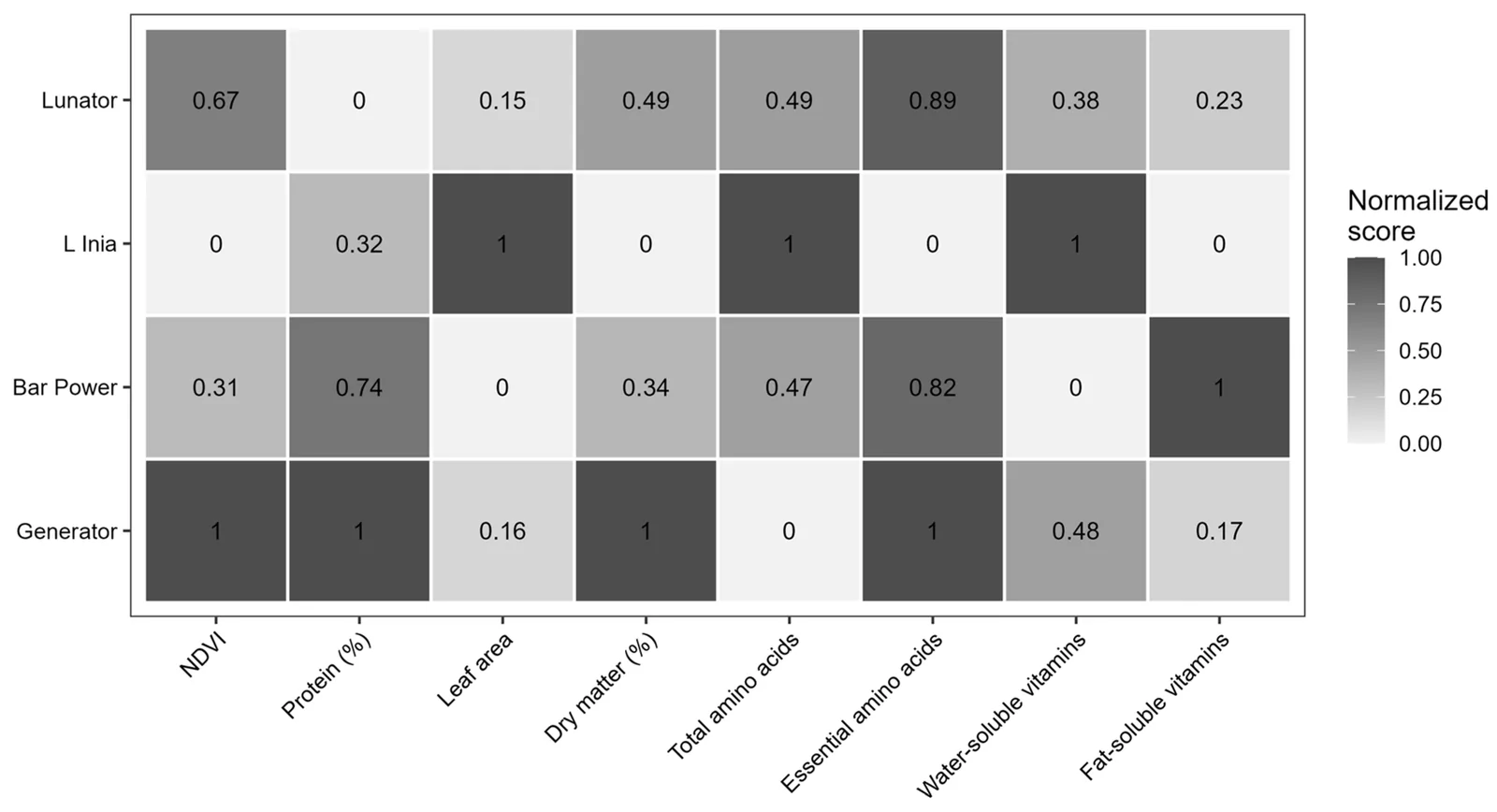
Multivariate characterization of rye genotypes based on normalized canopy, agronomic, and nutritional traits. Heatmap showing normalized values (0–1) for NDVI, crude protein concentration, leaf area, dry matter, total free amino acids, total essential free amino acids, total water-soluble vitamins, and total fat-soluble vitamins in four rye genotypes. Darker shading indicates higher relative values within each trait. Numbers within cells represent normalized values.

Integration of canopy, agronomic, protein, amino acid, and vitamin traits revealed distinct multivariate profiles among the four rye genotypes (Figure 6). Generator was characterized by comparatively high NDVI values, grain nitrogen and crude protein concentrations, and dry matter concentration. L-INIA was distinguished by greater plant height and leaf area and higher total water-soluble vitamin concentration, whereas Bar Power showed the highest total fat-soluble vitamin concentration together with higher concentrations of selected amino acids. Lunator generally occupied intermediate positions across the evaluated traits. Overall, the integrated visualization showed that genotype differentiation depended on the trait considered, with no single genotype exhibiting the highest values across all agronomic, canopy, and nutritional variables.

**Figure 7:**
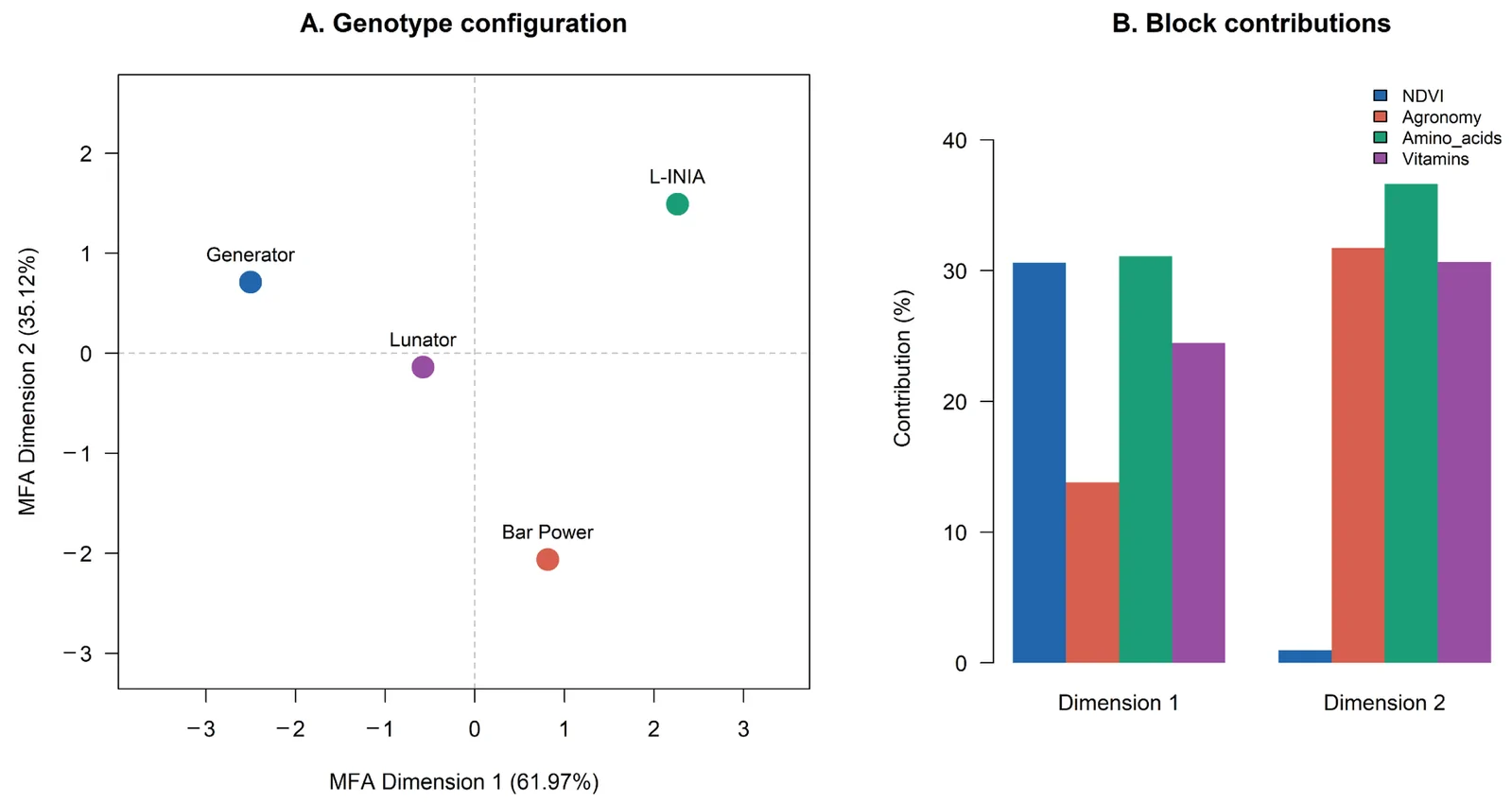
Exploratory multiple factor analysis (MFA) integrating canopy, agronomic, free amino acid, and vitamin traits in four rye genotypes. (**A**) Genotype configurations on the first two MFA dimensions, explaining 61.97% and 35.12% of the total inertia, respectively. (**B**) Relative contributions of the four variable blocks to each dimension. The analysis was performed using genotype-level means (n = 4), with three UAV-derived NDVI measurements, leaf area, 18 free amino acids, and seven vitamins. All variables were standardized, and blocks were weighted according to the inverse square root of their first eigenvalue. Genotype positions represent exploratory multivariate patterns rather than statistically confirmed separation.

To further examine the joint structure of canopy, agronomic, amino acid, and vitamin traits, an exploratory multiple factor analysis (MFA) was performed using genotype-level means from four data blocks. The first two dimensions accounted for 61.97% and 35.12% of the total inertia, respectively, explaining 97.09% cumulatively (Figure 7). Dimension 1 was primarily influenced by the amino acid (31.11%) and NDVI (30.61%) blocks, followed by vitamins (24.47%) and agronomic traits (13.81%). Dimension 2 was mainly associated with amino acids (36.63%), agronomic traits (31.74%), and vitamins (30.65%), whereas the NDVI block contributed only 0.98%. The MFA revealed distinct descriptive positions among the four genotypes. Generator and L-INIA occupied opposite sides of Dimension 1, while Bar Power was characterized by a negative position along Dimension 2. Lunator occupied a comparatively central position. At the individual-variable level, leaf area, NDVI at the three acquisition dates, and selected vitamins were prominent contributors to Dimension 1, whereas leaf area, vitamin C, and α-tocotrienol contributed strongly to Dimension 2 (Figure S3). These patterns describe multivariate variation among the four genotype means and should not be interpreted as statistically validated genotype separation.

**Figure 8:**
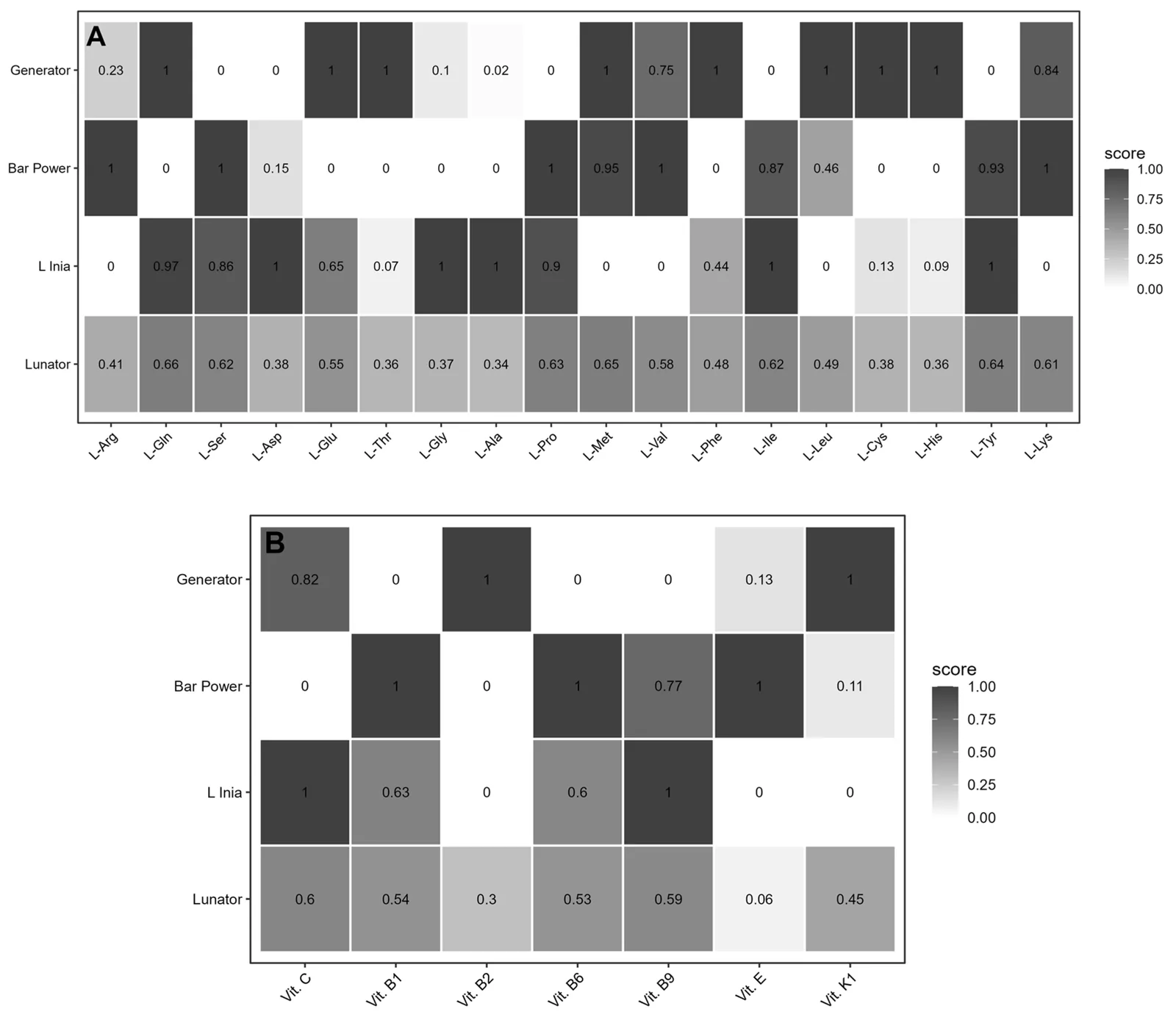
Comparative profiles of individual free amino acids and vitamins in rye genotypes. Heatmaps showing normalized values (0–1) for (**A**) individual free amino acids and (**B**) individual vitamins. In panel (**B**), the variable labeled “Vitamin E” corresponds specifically to α-tocotrienol, the only vitamin E isoform quantified in this study; other tocopherol and tocotrienol isoforms were not determined. Darker shading indicates higher relative concentrations within each compound across genotypes. Numbers within cells represent normalized values.

The genotype-specific patterns of individual free amino acids and vitamins are further illustrated in Figure 8A,B, respectively.

Although total free amino acid and total essential free amino acid concentrations were normalized for visualization purposes, these variables did not differ significantly among genotypes (*p* > 0.05). Therefore, differences in their normalized values should not be interpreted as statistically significant genotype effects. The variable labeled “Total fat-soluble vitamins” represents the combined concentrations of α-tocotrienol and vitamin K1 only.

## 3. Discussion

The present study revealed distinct genotype-specific patterns in temporal canopy development, plant architecture, grain protein concentration, free amino acid composition, and vitamin profiles under the environmental and management conditions evaluated. These differences highlight the relevance of genotypes when characterizing the nutritional phenotype of rye and indicate that substantial variation can occur among rye materials grown within a common field environment [28]. Although rye has traditionally been valued for its resilience, biomass production, and adaptation to marginal environments, its intraspecific nutritional diversity remains comparatively underexplored [29]. The present results therefore extend conventional agronomic characterization by showing that canopy, architectural, and grain nutritional traits provide complementary information for distinguishing rye genotypes. Importantly, however, these findings represent patterns observed during a single growing season and at one experimental location and should therefore be interpreted as genotype-specific characteristics under the conditions evaluated rather than as evidence of broad environmental stability.

One of the clearest patterns was the temporal differentiation in UAV-derived NDVI among genotypes. Generator consistently exhibited comparatively high NDVI values, particularly during the earlier and intermediate evaluations, whereas differences among genotypes became less pronounced by the final assessment. NDVI is widely related to canopy greenness and can reflect variation in canopy density, chlorophyll status, radiation interception, and photosynthetically active vegetation [30]. Accordingly, the temporal differences observed here indicate distinct patterns of canopy development among the evaluated genotypes rather than direct differences in physiological efficiency. This distinction is important because NDVI provides an indirect spectral characterization of the canopy and does not, by itself, identify the physiological mechanisms responsible for differences among genotypes. The progressive convergence of NDVI values toward the final evaluation further illustrates the value of repeated measurements across crop development. A single late-season assessment would have captured considerably less differentiation among the four genotypes than the temporal UAV surveys.

At the same time, canopy characteristics should not be interpreted as direct indicators of final grain nutritional composition [31]. Generator, for example, combined comparatively high NDVI values with the highest grain nitrogen (2.74%) and estimated crude protein concentration (approximately 15.96%), but did not exhibit the highest concentrations of all nutritional components. L-INIA showed the highest total water-soluble vitamin concentration, whereas Bar Power exhibited the highest total fat-soluble vitamin concentration. Thus, the genotype rankings differed depending on the trait considered, emphasizing that canopy development and grain nutritional composition represent complementary rather than interchangeable dimensions of genotype characterization.

UAV-derived NDVI is widely used as a rapid and non-destructive approach for characterizing crop canopies, particularly in applications related to biomass, nitrogen status, and crop development [32]. The present study demonstrates its usefulness for characterizing genotype-dependent temporal canopy dynamics. Generator exhibited the highest integrated NDVI, whereas L-INIA and Bar Power showed the greatest increases in NDVI between July and October. The exploratory correlation analysis identified several strong positive and negative rank associations between temporal NDVI descriptors and individual amino acids and vitamins. However, none of these associations reached statistical significance after correction for multiple comparisons. Importantly, Spearman coefficients approaching or reaching ±1.00 must be interpreted cautiously when calculated from only four genotype-level observations, because identical genotype rankings can produce apparently perfect correlations without providing statistically significant evidence of an underlying relationship. Furthermore, individual UAV measurements could not be reliably matched to their corresponding biochemical samples, preventing the evaluation of plot-level canopy–nutrition relationships. Consequently, these results do not establish NDVI as a predictor of grain protein, amino acid, or vitamin composition. Instead, they support the complementary value of temporal canopy monitoring and direct biochemical characterization for describing genotype-specific phenotypes [33]. Future investigations should incorporate larger genotype panels, multiple environments, and verified plot-level pairing between UAV and biochemical measurements to determine whether robust predictive relationships can be established.

An additional consideration is that NDVI represents only a portion of the spectral information available from multispectral imagery. Although NDVI was useful for characterizing temporal differences in canopy development among rye genotypes, its interpretation may be influenced by canopy architecture, illumination conditions, and background characteristics. In a multi-domain investigation of grape-cluster detection, Rana et al. [34] demonstrated that the relative performance of RGB and near-infrared imagery varied with image quality, spectral domain, and environmental complexity. Although their study addressed computer-vision-based object detection rather than vegetation-index estimation, it highlights the importance of considering spectral and acquisition-related variability when interpreting agricultural imagery. In the present study, the red-edge band was acquired but was not included in the NDVI-based analysis. Complementary indices incorporating this band, such as the normalized difference red-edge index (NDRE), could provide additional information on canopy spectral responses and potentially improve the characterization of genotype-dependent differences. Future research should evaluate these indices alongside NDVI and determine their relationships with grain nutritional traits using larger genotype panels and appropriately matched experimental observations.

The exploratory multiple factor analysis (MFA) provided an additional multivariate perspective beyond the independently normalized heatmaps by examining the joint structure of canopy, agronomic, amino acid, and vitamin traits. The first two dimensions explained 97.09% of the total inertia, with NDVI (30.61%) and amino acid traits (31.11%) contributing prominently to the first dimension, whereas amino acids (36.63%), agronomic traits (31.74%), and vitamins (30.65%) contributed most strongly to the second dimension. These patterns revealed contrasting multivariate profiles among the evaluated genotypes, particularly between Generator and L-INIA along the first dimension and for Bar Power along the second dimension. Nevertheless, the analysis was based on only four genotype-level observations, and the agronomic block comprised leaf area alone. Therefore, the resulting configurations should be interpreted as exploratory patterns of multivariate variation rather than evidence of statistically significant genotype separation or validated predictive relationships between canopy development and grain nutritional composition.

The agronomic measurements further demonstrated distinct plant architectural characteristics among the evaluated genotypes. L-INIA exhibited the greatest plant height and leaf area, with a mean leaf area of 31.0 cm^2^, nearly twice that observed in Generator (16.8 cm^2^), Bar Power (14.4 cm^2^), and Lunator (16.2 cm^2^). Greater leaf area can increase the photosynthetic surface available for radiation interception, although its contribution to crop performance depends on canopy structure, developmental stage, and environmental conditions [35]. Therefore, the larger leaf area observed in L-INIA should primarily be interpreted as a distinct architectural characteristic rather than direct evidence of greater photosynthetic capacity or agronomic superiority.

Differences in biomass allocation and plant architecture may be associated with trade-offs in the distribution of carbon and nitrogen among vegetative and reproductive organs [36]. Nevertheless, such allocation processes were not directly quantified in the present study. Consequently, the distinct combinations observed among plant architecture, dry matter, grain protein, and nutritional traits should be considered phenotypic patterns that warrant further physiological investigation rather than evidence of specific resource-allocation mechanisms. This distinction is particularly relevant for L-INIA, which combined greater plant height and leaf area with the highest total water-soluble vitamin concentration but lower grain protein concentration than Generator and Bar Power.

Generator showed the highest grain nitrogen and estimated crude protein concentrations, reaching 2.74% and approximately 15.96%, respectively, compared with 2.24% nitrogen and approximately 12.97% crude protein in Lunator. Grain protein concentration in cereals is influenced by nitrogen acquisition, remobilization, and grain-filling processes [37,38]. However, because these processes were not directly measured in the present experiment, the higher grain protein concentration observed in Generator cannot be attributed to a specific nitrogen-use mechanism. Instead, the results identify Generator as the genotype with the highest grain nitrogen and crude protein concentrations under the evaluated conditions. This distinction is important because it preserves the agronomic relevance of the observed differences without inferring unmeasured physiological processes or broader genotype stability.

Interestingly, differences in crude protein concentration were not accompanied by significant differences in the total free amino acid pool or the total essential free amino acid pool. Total free amino acid concentrations remained within a comparatively narrow range of approximately 9500–9800 µg g^−1^ DW, despite significant differences in crude protein concentration. This apparent contrast should be interpreted cautiously because crude protein and free amino acids represent different nitrogen-containing fractions of the grain. Crude protein is estimated from total nitrogen using a conversion factor, whereas the free amino acid pool represents only the non-protein-bound amino acid fraction. Consequently, the two measurements are not expected to vary proportionally.

Despite the similarity in total free amino acid concentrations, individual amino acids differed significantly among genotypes. Although genotype-dependent differences were detected for individual free amino acids, the significance of pairwise comparisons varied among compounds. For example, Generator exhibited the highest numerical concentrations of glutamate and leucine, but significant differences were observed only relative to Bar Power and L-INIA, respectively. Similarly, L-INIA and Bar Power exhibited comparable tyrosine concentrations without a significant difference between them. These findings indicate that genotype-specific amino acid patterns should be interpreted at the level of individual pairwise comparisons rather than as evidence of consistently superior concentrations across all genotypes. In contrast, lysine did not differ significantly among genotypes. Given the unusually high contribution of free lysine to the total free amino acid pool, the chromatographic assignment of lysine was re-examined by inspection of representative RP-HPLC-DAD chromatograms and comparison with the amino acid standard mixture. This review supported the retention-time-based identification of lysine and did not reveal evident chromatographic co-elution in the corresponding region. Nevertheless, because no orthogonal confirmation method was applied, these results should be interpreted in light of the analytical specificity of the FMOC-based HPLC-DAD procedure used in this study. These compositional differences may be nutritionally relevant because individual essential amino acids can influence the nutritional value of cereal grains. Lysine is particularly relevant because it is commonly limiting in cereals, whereas sulfur-containing amino acids such as methionine and cysteine are also important components of cereal-based diets [39]. Accordingly, characterization of individual amino acids provides information that is not captured by crude protein concentration alone. Nevertheless, the present results should be considered evidence of genotype-specific compositional differences under the evaluated conditions rather than proof of superior nutritional value for a particular genotype.

Vitamin composition provided an additional dimension of differentiation among the evaluated rye genotypes. L-INIA exhibited the highest total water-soluble vitamin concentration (223 mg 100 g^−1^ DW), whereas Bar Power showed the highest total fat-soluble vitamin concentration (81 µg g^−1^ DW), nearly twice the values observed in the remaining genotypes. Generator and Lunator showed intermediate total vitamin concentrations, although differences were also evident for individual vitamins. These results indicate that evaluation of total vitamin pools alone may overlook relevant genotype-specific compositional differences. This is particularly relevant because cereals are not always considered in terms of genotype-dependent vitamin composition, despite the nutritional importance of B-complex vitamins, tocopherols, and phylloquinones in grain quality and human nutrition [40].

The distinct vitamin profiles observed among genotypes may reflect differences in the metabolic processes involved in vitamin biosynthesis, accumulation, and storage. For example, variation in ascorbate and folate concentrations may arise from genotype-dependent regulation of their respective metabolic pathways [41]. However, the present study did not evaluate gene expression, enzyme activity, metabolic fluxes, or other mechanistic determinants of vitamin accumulation. Therefore, the higher concentrations of water-soluble vitamins observed in L-INIA should not be attributed directly to its greater leaf area or presumed photosynthetic capacity, nor should the higher fat-soluble vitamin concentration of Bar Power be interpreted as evidence of enhanced isoprenoid or lipid-associated metabolism. Instead, these results identify distinct vitamin profiles that warrant further investigation to determine the physiological and molecular mechanisms underlying their variation. An additional methodological consideration concerns the potential degradation of thermolabile vitamins during sample preparation. Grain samples were dried in a forced-air oven at 60 °C for 48 h before chromatographic analysis, a procedure that may have affected the stability and recovery of heat-sensitive compounds, particularly ascorbic acid (vitamin C), as well as certain B-complex vitamins. Thermal exposure and oxidation during prolonged drying may result in partial vitamin degradation, potentially leading to an underestimation of their original concentrations in the grain. Although all genotypes were subjected to the same drying conditions, possible genotype-dependent differences in vitamin stability or matrix composition cannot be excluded. Furthermore, vitamin retention during drying was not experimentally evaluated in this study. Therefore, the reported concentrations should be interpreted as those measured in the processed grain samples rather than as definitive estimates of the original vitamin content before drying. Future studies should consider low-temperature drying or freeze-drying procedures and direct assessments of vitamin retention to minimize and quantify potential processing-related losses.

When the different groups of traits were considered together, no single genotype exhibited the highest values across all canopy, agronomic, and nutritional variables. Generator combined comparatively high NDVI values with the highest grain nitrogen and crude protein concentrations and the highest dry matter concentration. L-INIA was characterized by greater plant height and leaf area and the highest total water-soluble vitamin concentration, whereas Bar Power showed the highest total fat-soluble vitamin concentration together with higher concentrations of selected free amino acids. Lunator generally exhibited intermediate values across the evaluated traits. The normalized multivariate visualization therefore illustrates distinct genotype-specific profiles rather than an overall ranking of performance. Importantly, normalized values in this analysis represent relative differences within individual traits and should not be interpreted as evidence that a particular genotype is universally superior.

This multivariate perspective reinforces one of the central implications of the study: canopy, agronomic, and nutritional measurements capture different aspects of genotype characterization. The temporal UAV measurements were useful for distinguishing canopy developmental patterns, particularly during earlier and intermediate assessments, whereas direct biochemical analyses identified differences in grain protein, individual free amino acids, and vitamins that could not be inferred from canopy information alone. Consequently, the value of combining these approaches lies in their complementarity rather than in using NDVI as a surrogate for grain nutritional composition. This distinction also provides a more appropriate framework for future applications, in which spectral traits could be integrated with direct nutritional measurements and subsequently evaluated for predictive capacity using larger genotype panels and independent validation datasets.

The present findings should also be interpreted in light of the experimental scope. The study was conducted at a single location during one growing season and included four rye genotypes, with the UAV-based NDVI assessment conducted under two closely spaced sowing dates. The two sowing dates introduced variation in establishment timing within the same field environment but do not constitute independent environmental replication. Consequently, the present experiment does not allow conclusions regarding genotype × environment interactions, multi-environment stability, or broad adaptation of the evaluated genotypes. Similarly, the relatively small genotype panel limits extrapolation to the wider genetic diversity of rye. These limitations do not invalidate the observed differences among genotypes under the conditions evaluated, but they define the appropriate scope of inference. Validation across multiple growing seasons, locations, and larger germplasm panels will therefore be required to determine the stability and broader relevance of the observed canopy and nutritional profiles.

Within this defined scope, the results contribute to the broader characterization of rye as a crop with potentially valuable agronomic and nutritional variation. Rye is already recognized for its tolerance to cold conditions, adaptation to low-input production systems, and contribution to soil protection [42]. Characterizing variation in grain nutritional traits alongside conventional agronomic and canopy measurements may broaden the criteria considered when evaluating rye germplasm for different end uses. Future studies should therefore extend this framework across environments, incorporate yield and additional agronomic components, and investigate the physiological and molecular bases underlying variation in free amino acid and vitamin composition. Larger datasets would also make it possible to test whether multispectral or other remote-sensing variables can reliably predict specific grain nutritional attributes, rather than assuming such relationships from canopy characteristics alone.

Overall, the present study demonstrates that the four rye genotypes exhibited distinct canopy developmental patterns, plant architecture, grain nitrogen and crude protein concentrations, free amino acid composition, and vitamin profiles under the environmental and management conditions evaluated. Rather than identifying a universally superior genotype, the integrated characterization revealed trait-dependent differences among materials. Temporal UAV-derived NDVI provided complementary information on canopy development, while direct biochemical analyses were necessary to characterize grain nutritional composition. These findings support an integrated approach to rye characterization while emphasizing the need for multi-environment validation before conclusions regarding genotype stability, broad adaptation, or nutritional superiority can be established.

## 4. Materials and Methods

### 4.1. Experimental Design

Field experiments were conducted during the 2025 growing season at the Maquehue Experimental Field, Facultad de Ciencias Agropecuarias y Medioambiente, Universidad de La Frontera, Freire, Chile. Four rye (*Secale cereale* L.) genotypes developed by Universidad de La Frontera were evaluated: Generator, Bar Power, L-INIA, and Lunator. Prior to sowing, weed control was performed in February 2025 using paraquat at 3 L ha^−1^. The soil was subsequently prepared with a rotary tiller to obtain a uniform seedbed free of residual vegetation. Sowing was carried out manually using a continuous-flow seeder at a target density of 400 plants m^−2^. Seeds were previously treated with the fungicide Indar Flo at 200 mL per 100 kg of seed. Fertilization was based on local agronomic recommendations. At sowing, the full phosphorus dose was applied in the seed furrow (138 kg ha^−1^ P_2_O_5_ equivalent). After crop emergence, additional nutrients were supplied at rates of 90 kg ha^−1^ K_2_O, 75 kg ha^−1^ Mg, and 75 kg ha^−1^ S. Nitrogen fertilization was applied at a total rate of 150 kg N ha^−1^ using urea as the nitrogen source. The nitrogen dose was split into multiple applications, with the first application performed during vegetative growth, before the first cutting, and subsequent applications carried out after each cutting. The experiment was established as a randomized complete block design with four replications. Each plot consisted of seven rows spaced 0.17 m apart and 4.0 m in length, corresponding to a nominal planted area of 4.76 m^2^. Two sowing dates were evaluated for each genotype: 27 March and 31 March 2025. Both sowing dates were included in the UAV-based NDVI monitoring, whereas all destructive agronomic measurements and grain compositional analyses were conducted exclusively on plots established on 27 March 2025. Agronomic measurements, including plant height, leaf area, fresh biomass, and dry matter, were conducted during the first week of August 2025, corresponding to the advanced tillering–early stem elongation stage (BBCH 29–31). Sampling was scheduled when the experimental crop canopy reached an approximate height of 40 cm, which was used as a general field reference rather than a fixed height criterion for each genotype. Consequently, individual genotype heights varied around this reference value. Biomass was manually harvested using scissors from a 0.6 m^2^ quadrat positioned in the central area of each plot to minimize border effects. The harvested material was subsequently used for fresh biomass, dry matter, and complementary agronomic determinations.

### 4.2. Field Measurements and Leaf Sampling

Leaf samples were collected from each experimental plot for agronomic measurements. For each experimental replicate, ten healthy, fully expanded leaves were randomly collected from the upper canopy during the first week of August 2025, corresponding to the advanced tillering–early stem elongation stage (BBCH 29–31). Leaves were selected from comparable positions within the canopy to minimize variability associated with leaf developmental stage and canopy position. Samples were placed in paper bags, transported to the Applied Entomology Laboratory at Universidad de La Frontera, and stored at −20 °C until processing.

#### 4.2.1. Leaf Area Determination

Leaf area was determined using the ten fully expanded upper-canopy leaves collected from each experimental unit during the first week of August 2025, as described in Section 4.2. Leaves were individually scanned at high resolution and analyzed using ImageJ software v1.42j (National Institutes of Health, Bethesda, MD, USA). Results were expressed as cm^2^ leaf^−1^ [43].

#### 4.2.2. Fresh and Dry Matter Determination

Fresh biomass collected from the harvest quadrat was immediately weighed to determine fresh weight (FW). Representative subsamples were cut into small fragments, placed in aluminum trays, and dried in a forced-air oven at 60 °C for 48–72 h until constant weight was achieved. Samples were subsequently cooled in a desiccator and reweighed to determine dry weight (DW) [44]. Fresh biomass was recorded within a 0.6 m^2^ harvest quadrat and expressed as t ha^−1^ after area-based conversion.

Dry matter percentage (DM) was calculated according to Equation (1):

DM% = ((Dry Weight)/(Fresh Weight)) × 100(1)

where DW is the oven-dried sample mass and FW is the initial fresh sample mass [45].

#### 4.2.3. Climatic Parameters

Meteorological variables during the experimental period were obtained from the Maquehue weather station, Universidad de La Frontera, Freire, La Araucanía Region, Chile. Recorded variables included mean air temperature (°C), relative humidity (%), and cumulative precipitation (mm). These data were used to characterize environmental conditions during crop establishment and development (Table 3).

To provide a comprehensive characterization of the environmental conditions throughout the experimental period, monthly mean air temperature, mean relative humidity, and cumulative precipitation were also compiled for the entire growing season, from March 2025 to February 2026 (Table S6). The detailed daily climatic records for March 2025 (Table S5) were retained to characterize the specific environmental conditions during crop establishment.

### 4.3. Grain Nitrogen and Protein Determination

Grain was harvested in early February 2026, when plants exhibited dry spikes and hard grains. The entire plot (seven 4.0-m rows) was harvested, including border rows and row ends. After harvest, grain from each plot was thoroughly homogenized, and a representative 500-g subsample was collected and transported to the laboratory for compositional analyses. Samples were dried in a forced-air oven at 60 °C for 48 h and subsequently ground to a particle size of 0.2 mm using a laboratory mill. For nitrogen determination, approximately 70 mg of dried and ground sample was weighed into analytical capsules and analyzed using a DUMATHERM^®^ N Pro nitrogen analyzer (Gerhardt Analytical Systems, Königswinter, Germany) based on the combustion method. Grain crude protein concentration was estimated from total nitrogen concentration using the rye-specific nitrogen-to-protein conversion factor of 5.83 (Jones factor), rather than the general conversion factor of 6.25. Nitrogen and crude protein concentrations were expressed as percentages of dry weight (% DW) [46,47].

### 4.4. Amino Acid and Vitamin Analyses

Compositional analyses were performed on three independent biological replicates per genotype. Each replicate consisted of grain independently harvested from a different experimental plot (block) established on 27 March 2025. Samples were dried at 60 °C, milled to a particle size of 0.2 mm, and stored in airtight containers until analysis.

#### 4.4.1. Free Amino Acid Determination by RP-HPLC-DAD

Free amino acids were extracted using an acid-assisted procedure with minor modifications. Briefly, 0.50 g of dried and ground grain sample was transferred to a 15 mL centrifuge tube and mixed with 5 mL of 0.1 N HCl. Samples were sonicated for 30 min at 37 kHz in an ultrasonic bath (Branson 2510, Branson Ultrasonics Corp., Danbury, CT, USA), while maintaining the temperature between 20 and 30 °C. Extracts were centrifuged at 4000 rpm for 10 min (Eppendorf 5810 R, Eppendorf, Hamburg, Germany), and the resulting supernatants were filtered through 0.22 µm hydrophilic PVDF syringe filters (MilliporeSigma, Burlington, MA, USA). Prior to derivatization, extracts were diluted five-fold with the extraction solvent. For pre-column derivatization, 100 µL of extract or standard solution was mixed with 200 µL of 0.5 M sodium borate buffer (pH 8.6) and 400 µL of 6 mM fluorenylmethoxycarbonyl chloride (FMOC-Cl) in acetonitrile. After incubation for 10 min at room temperature, 300 µL of 12.5 mM 1-adamantylamine (ADAM) prepared in water:acetonitrile (1:3, *v*/*v*) was added to terminate the derivatization reaction. Samples were centrifuged at 10,000× *g* for 5 min and transferred to HPLC vials. Chromatographic analysis was performed using an LC-2050 liquid chromatograph (Shimadzu Corporation, Kyoto, Japan) equipped with a photodiode array detector (PDA). Separation was achieved using a Poroshell EC-C18 Isis column (3 × 100 mm, 2.7 µm) maintained at 25 °C. Mobile phase A consisted of 50 mM sodium acetate adjusted to pH 4.15 with phosphoric acid, whereas mobile phase B consisted of acetonitrile. The flow rate was 0.7 mL min^−1^, and the injection volume was 10 µL. Gradient elution was programmed as follows: 0–4 min, 85% A and 15% B; 4–16 min, 77% A and 23% B; 16–21 min, 62% A and 38% B; 21–27 min, 60% A and 40% B; and 27–33 min, 85% A and 15% B. Detection was performed at 265 nm. Quantification was performed using external calibration curves prepared from a certified amino acid standard mixture (Sigma-Aldrich AAS18-5ML, Sigma-Aldrich, St. Louis, MO, USA), with standard concentrations ranging from 2.5 to 80 µM. Free amino acid concentrations were expressed as µg g^−1^ DW [48]. Peak identification was performed by comparing the retention times of sample peaks with those of the amino acid standard mixture analyzed under the same chromatographic conditions. The chromatographic profile was re-examined to verify the peak assigned to lysine, given its high relative abundance in the analyzed samples (Figure S4). Under the chromatographic conditions used, the lysine peak was identified as a distinct resolved signal based on retention-time matching, and no evident peak distortion or visual indication of major co-elution was observed in the corresponding retention window. The analytical method was evaluated for linearity, limits of detection (LOD) and quantification (LOQ), recovery, and precision. Calibration curves for all 18 free amino acids exhibited coefficients of determination (R^2^) of 0.9999. The analytical recovery ranged from 94.46% to 97.94%, while the coefficient of variation (CV) was 2.0%. Analyte-specific LOD and LOQ values, together with the corresponding recovery and precision parameters, are presented in Supplementary Table S7.

#### 4.4.2. Water-Soluble Vitamin Determination by HPLC-DAD

Water-soluble vitamins were extracted from 500 mg of dried and powdered grain sample using 4 mL of methanol:water (80:20, *v*/*v*). Samples were sonicated for 30 min, centrifuged at 2000 rpm for 5 min, and filtered through 0.22 µm PVDF membranes. Vitamin analysis was performed using an LC-2050C 3D HPLC system (Shimadzu Corporation, Kyoto, Japan) equipped with a quaternary pump, autosampler, column oven, and photodiode array detector. Separation was achieved using an EC NUCLEODUR^®^ C18 Isis column (250 × 4.6 mm, 5 µm; Macherey-Nagel GmbH & Co. KG, Düren, Germany) protected by a compatible guard column. The column temperature was maintained at 40 °C, and the injection volume was 20 µL. Mobile phases consisted of solvent A (0.1% trifluoroacetic acid in ultrapure water) and solvent B (acetonitrile), using a gradient elution program optimized for vitamin separation. Quantified compounds included vitamin C (ascorbic acid), vitamin B1 (thiamine), vitamin B2 (riboflavin), vitamin B6 (pyridoxine), and vitamin B9 (folic acid). External calibration curves were prepared using ten concentration levels in methanol:water (80:20, *v*/*v*). Method recoveries ranged from 78 to 93%, with coefficients of variation below 5%. Water-soluble vitamin concentrations were expressed as mg 100 g^−1^ DW [49].

#### 4.4.3. Fat-Soluble Vitamin Determination by HPLC-DAD

Fat-soluble vitamins were determined after alkaline saponification followed by hexane extraction. Briefly, 0.50 g of dried and ground grain sample was mixed with 7.5 mL of 50% (*w*/*v*) KOH containing 0.1% butylated hydroxytoluene (BHT) as an antioxidant. Samples were vortexed for 30 s and saponified in an ultrasonic bath at 60 °C for 45 min. After cooling, 7.5 mL of hexane was added, and samples were vigorously shaken for 3 min. The mixture was centrifuged at 4000 rpm for 5 min, and the organic phase was recovered. Hexane extraction was repeated twice, and the combined organic fractions were washed with ultrapure water and saturated NaCl solution. The organic extracts were subsequently concentrated under reduced pressure at 40 °C using a rotary evaporator (Büchi Rotavapor R-210, BÜCHI Labortechnik AG, Flawil, Switzerland). The residue was reconstituted in 2 mL of methanol, filtered through 0.22 µm membranes, and injected into the HPLC system. Chromatographic analysis was performed using a MultoKrom C18 column (250 × 4.6 mm, 5 µm; Merck Millipore, Darmstadt, Germany) maintained at 30 °C. The injection volume was 20 µL. The mobile phase consisted of acetonitrile:methanol (98:2, *v*/*v*) under isocratic conditions at a flow rate of 1.0 mL min^−1^. Detection wavelengths were 294 nm for α-tocotrienol and 246 nm for vitamin K1. Quantification was performed by external calibration using α-tocotrienol as the reference standard, with stock solutions prepared at 1000 mg L^−1^ in methanol containing 0.1% BHT. Only α-tocotrienol was quantified as a representative form of vitamin E; other tocopherol and tocotrienol isomers were not individually quantified. Accordingly, the reported α-tocotrienol concentrations should not be interpreted as total vitamin E content. Fat-soluble vitamin concentrations were expressed as µg g^−1^ DW [50]. The analytical performance of the HPLC-DAD method was evaluated for α-tocotrienol and vitamin K1. Calibration curves for both compounds exhibited coefficients of determination (R^2^) of 0.9999. Analytical recoveries were 96.26% for α-tocotrienol and 92.88% for vitamin K1, with a coefficient of variation (CV) of 3.0% for both analytes.

### 4.5. UAV Flights and Multispectral Image Analysis

Canopy monitoring was performed using an unmanned aerial vehicle (UAV) equipped with a Phantom 4 Multispectral platform (DJI, Shenzhen, China). A total of three multispectral surveys were conducted on 29 July, 3 September, and 23 October 2025 to characterize temporal changes in canopy spectral characteristics during crop development. Flights were conducted under suitable weather conditions, with low wind speed and absence of rainfall. Images were acquired at 50 m above ground level, resulting in an approximate spatial resolution of 8 cm pixel^−1^. The multispectral sensor simultaneously captured reflectance in the green (550 nm), red (660 nm), red-edge (735 nm), and near-infrared (790 nm) bands, in addition to RGB imagery. Orthomosaics were generated using DJI Terra v4.2 (DJI, Shenzhen, China), and subsequent spectral layer processing and analysis were performed using QGIS v3.40 (Bratislava) (QGIS Development Team, Open Source Geospatial Foundation, Zurich, Switzerland). Radiometric calibration was performed using the downwelling light sensor (DLS) integrated into the Phantom 4 Multispectral platform, together with calibrated reflectance panel measurements acquired during the flight campaigns. These procedures were used to compensate for variations in incident solar irradiance and improve radiometric consistency among acquisition dates. Experimental plot boundaries were delineated in QGIS, and a 25 cm inward buffer was applied to each plot to minimize spectral contamination from adjacent plots and boundary pixels. Soil and shadow pixels were identified and excluded using spectral threshold-based masking in QGIS prior to NDVI extraction. Vegetation pixels were retained on the basis of their spectral response, whereas non-vegetated and shaded pixels were excluded to minimize the influence of soil background and shadow effects on plot-level NDVI estimates. Plot-level NDVI values were subsequently extracted from the masked multispectral orthomosaics for each acquisition date. The normalized difference vegetation index (NDVI) was calculated for each experimental plot according to Equation (2):

NDVI = ((NIR − Red)/(NIR + Red))(2)

where NIR represents near-infrared reflectance and Red the red-band reflectance. NDVI values typically range from −1 to 1, with higher positive values generally indicating greater canopy greenness and vegetation density [51].

### 4.6. Statistical Analysis

All statistical analyses were performed using R software v4.3.2 (R Foundation for Statistical Computing, Vienna, Austria). Data were assessed for normality and homogeneity of variances using the Shapiro–Wilk and Levene tests, respectively. Agronomic traits and grain nitrogen and crude protein concentrations, obtained exclusively from plots established on 27 March 2025, were analyzed using one-way analysis of variance (ANOVA), with genotype as the fixed factor and four independent experimental replicates per genotype (n = 4). Free amino acid and vitamin concentrations, also obtained exclusively from plots established on 27 March 2025, were analyzed separately using one-way ANOVA, with genotype as the fixed factor and three independent biological replicates per genotype (n = 3), each originating from a different experimental plot (block). For the biochemical characterization of free amino acids and vitamins, three of the four experimental blocks were randomly selected before laboratory analysis, using the same selected blocks for all four genotypes. This subsampling strategy was adopted due to laboratory analytical capacity constraints while maintaining a balanced experimental structure with three independent biological replicates per genotype (n = 3). The selection was performed independently of the biochemical results to minimize potential selection bias. To account for multiple testing across the 18 free amino acids and seven vitamins, *p*-values obtained from the individual ANOVAs were adjusted using the Benjamini–Hochberg procedure to control the false discovery rate (FDR) at 5%. The adjusted *p*-values were used to evaluate the statistical significance of genotype effects across individual compounds. Tukey’s honestly significant difference (HSD) test was used for pairwise genotype comparisons within each compound. Temporal NDVI data, which included plots from both sowing dates (27 and 31 March 2025), were analyzed using a linear mixed-effects model to account for repeated measurements of the same experimental plots across sampling dates. Genotype, sowing date, sampling date, and their interactions were included as fixed effects, whereas plot was included as a random effect to account for repeated observations of each experimental unit over time. Fixed effects were evaluated using Type III analysis of variance with Satterthwaite’s approximation for denominator degrees of freedom. Post hoc comparisons among genotypes within each sowing date × sampling date combination were performed using estimated marginal means followed by pairwise comparisons with Tukey adjustment at *p* ≤ 0.05. Results are presented as means ± standard error (SE). For multivariate visualization, trait means were normalized independently using min–max scaling to a 0–1 range. Normalized values were used to generate heatmaps describing relative genotype profiles for canopy, agronomic, and nutritional traits, as well as individual free amino acids and vitamins. These normalized values were used exclusively for visualization and were not interpreted as an overall performance index or genotype ranking. Figures were generated using the ggplot2, dplyr, tidyr, pheatmap, lme4, lmerTest, and emmeans packages within the R environment. Only UAV-derived NDVI observations from the 27 March sowing were included in the exploratory analyses relating canopy dynamics to grain nutritional composition. NDVI measurements from the second sowing date were excluded. Because individual UAV plots could not be reliably matched to the corresponding biochemical replicates, associations were evaluated using genotype-level means (n = 4), without assuming plot-level correspondence.

An exploratory multiple factor analysis (MFA) was conducted to investigate the joint multivariate structure of canopy, agronomic, and grain nutritional traits. Four quantitative data blocks were defined: UAV-derived NDVI at three acquisition dates, leaf area, 18 individual free amino acids, and seven individual vitamins. Genotype-level means were used as the common observational units (n = 4). Variables were centered and scaled to unit variance, and each block was weighted by the inverse square root of its first principal component eigenvalue before singular value decomposition of the combined matrix. The relative contributions of blocks and individual variables to each dimension were calculated from the squared component loadings. The MFA was implemented in base R. Because the analysis included only four genotype-level observations, it was interpreted as exploratory and descriptive, without inferential testing of genotype separation or assumptions of plot-level matching.

To examine the relationship between temporal canopy dynamics and grain nutritional composition, an additional exploratory analysis was conducted using UAV-derived NDVI data exclusively from the 27 March 2025 sowing. Genotype-level mean NDVI values were calculated for each acquisition date (29 July, 3 September, and 23 October 2025). Temporal descriptors included NDVI changes between consecutive acquisitions and over the complete monitoring period, the overall temporal slope, and the area under the NDVI curve (AUC). The slope was calculated as the difference between the final and initial mean NDVI divided by the 86-day monitoring period. AUC was calculated by trapezoidal integration using the actual intervals between acquisitions (36 and 50 days), and normalized AUC was obtained by dividing AUC by 86 days. Associations between temporal NDVI descriptors and grain nutritional composition were assessed using Spearman’s rank correlation coefficients. Nutritional variables included crude protein, 18 individual free amino acids, and seven vitamins. Because verified identifiers linking individual UAV observations with the corresponding biochemical samples were unavailable, the analyses were conducted using genotype-level means (n = 4), without assuming plot-level correspondence. Exact two-sided permutation *p*-values were obtained by enumerating all 24 possible permutations of the four genotype observations. *p*-values were adjusted using the Benjamini–Hochberg procedure across the 234 exploratory comparisons. Associations were considered statistically significant at an adjusted *p*-value below 0.05. These analyses were interpreted as exploratory and were not used to establish predictive relationships.

## 5. Conclusions

This study revealed distinct genotype-specific patterns in canopy development, agronomic traits, grain nitrogen and crude protein concentrations, free amino acid composition, and vitamin profiles in rye under the environmental and management conditions evaluated. UAV-derived NDVI detected temporal differences in canopy characteristics among genotypes, particularly during early and intermediate assessments, supporting its use as a rapid and non-destructive tool for monitoring canopy development. Generator exhibited comparatively high NDVI values and the highest grain nitrogen and crude protein concentrations, whereas L-INIA was characterized by greater plant height and leaf area and the highest total water-soluble vitamin concentration. Bar Power showed the highest total fat-soluble vitamin concentration and higher concentrations of selected free amino acids, while Lunator generally exhibited intermediate values across the evaluated traits. Overall, no single genotype showed the highest values across all canopy, agronomic, and nutritional variables, demonstrating that genotype differentiation was trait-dependent. The integration of temporal UAV-based canopy monitoring with direct grain nutritional profiling therefore provides complementary information for rye characterization rather than establishing NDVI as a surrogate for nutritional composition. Consistent with this interpretation, exploratory correlations between temporal NDVI descriptors and grain nutritional traits were not statistically significant after multiple-testing correction. These analyses were limited to four genotype-level observations and therefore cannot establish plot-level or predictive relationships. Given that the study was conducted at a single location during one growing season and included four genotypes, the observed patterns should be interpreted within the conditions evaluated. Future studies involving larger germplasm panels, multiple locations and growing seasons, and additional agronomic and molecular measurements will be necessary to assess the stability of these traits and determine their potential value for rye breeding and crop improvement.

## Figures and Tables

**Table 1:** Free amino acid composition (µg g^−1^ DW) of grains from four rye genotypes (Generator, Bar Power, L-INIA, and Lunator) evaluated under field conditions. Values are presented as mean ± standard error (SE). Different lowercase letters within the same row indicate significant differences among genotypes according to Tukey’s honestly significant difference (HSD) test (*p* ≤ 0.05). Values sharing at least one letter are not significantly different. DW, dry weight.

Compound	Generator	Bar Power	L-INIA	Lunator
L-Arg	137.33 ± 2.41 b	166.70 ± 2.85 a	128.42 ± 2.34 b	144.09 ± 11.58 ab
L-Gln	289.35 ± 5.05 a	230.30 ± 3.99 b	287.77 ± 5.00 a	269.10 ± 19.41 ab
L-Ser	254.88 ± 4.48 b	374.75 ± 6.51 a	357.68 ± 6.27 a	329.00 ± 37.44 ab
L-Asp	19.92 ± 0.35 b	21.29 ± 0.37 b	29.28 ± 0.52 a	23.49 ± 2.92 ab
L-Glu	17.46 ± 0.31 a	2.32 ± 0.04 b	12.09 ± 0.21 ab	10.62 ± 4.43 ab
L-Thr	157.44 ± 2.74 a	57.95 ± 1.00 b	64.75 ± 1.13 b	93.36 ± 32.09 ab
L-Gly	20.22 ± 0.36 b	19.04 ± 0.33 b	30.79 ± 0.53 a	23.35 ± 3.73 ab
L-Ala	749.78 ± 12.98 b	743.58 ± 12.88 b	1046.03 ± 18.53 a	846.31 ± 99.64 ab
L-Pro	261.95 ± 4.57 b	383.95 ± 6.67 a	372.25 ± 6.39 a	339.30 ± 38.85 ab
L-Met	147.70 ± 2.60 a	145.89 ± 2.53 a	114.77 ± 1.99 b	136.10 ± 10.68 ab
L-Val	130.69 ± 2.26 a	172.79 ± 2.99 a	1.36 ± 0.03 b	101.61 ± 51.58 ab
L-Phe	84.96 ± 1.48 a	77.37 ± 1.34 b	80.69 ± 1.41 ab	81.00 ± 2.19 ab
L-Ile	197.58 ± 3.42 b	219.99 ± 3.81 ab	223.43 ± 3.89 a	213.66 ± 8.10 ab
L-Leu	20.65 ± 0.36 a	10.52 ± 0.18 ab	1.76 ± 0.03 b	10.97 ± 5.45 ab
L-Cys	59.28 ± 1.03 a	24.59 ± 0.43 b	29.27 ± 0.52 b	37.71 ± 10.87 ab
L-His	63.42 ± 1.10 a	43.52 ± 0.76 b	45.23 ± 0.79 b	50.72 ± 6.37 ab
L-Tyr	26.36 ± 0.46 b	158.33 ± 2.74 a	167.92 ± 2.94 a	117.52 ± 45.67 ab
L-Lys	6912.10 ± 119.72 a	6932.80 ± 120.08 a	6806.09 ± 118.90 a	6883.27 ± 39.64 a

L-Arg = L-arginine; L-Gln = L-glutamine; L-Ser = L-serine; L-Asp = L-aspartic acid; L-Glu = L-glutamic acid; L-Thr = L-threonine; L-Gly = glycine; L-Ala = L-alanine; L-Pro = L-proline; L-Met = L-methionine; L-Val = L-valine; L-Phe = L-phenylalanine; L-Ile = L-isoleucine; L-Leu = L-leucine; L-Cys = L-cysteine; L-His = L-histidine; L-Tyr = L-tyrosine; L-Lys = L-lysine.

**Table 2:** Vitamin composition of grains from four rye genotypes (Generator, Bar Power, L-INIA, and Lunator) evaluated under field conditions. Water-soluble vitamins (C, B1, B2, B6, and B9) are expressed as mg 100 g^−1^ DW, whereas fat-soluble vitamins (E and K1) are expressed as µg g^−1^ DW. Values are presented as mean ± standard error (SE). Different lowercase letters within the same row indicate significant differences among genotypes according to Tukey’s honestly significant difference (HSD) test (*p* ≤ 0.05). Values sharing at least one letter are not significantly different. DW, dry weight.

Vitamin	Unit	Generator	Bar Power	L-INIA	Lunator
Vit. C	mg 100 g^−1^ DW	190.42 ± 3.33 a	128.65 ± 2.20 b	204.36 ± 3.44 a	174.42 ± 23.27 ab
Vit. B1	mg 100 g^−1^ DW	0.23 ± 0.01 b	6.96 ± 0.14 a	4.45 ± 0.10 ab	3.88 ± 1.96 ab
Vit. B2	mg 100 g^−1^ DW	0.08 ± 0.01 a	0.00 ± 0.00 b	0.00 ± 0.00 b	0.03 ± 0.03 ab
Vit. B6	mg 100 g^−1^ DW	0.11 ± 0.01 b	2.91 ± 0.07 a	1.79 ± 0.04 ab	1.60 ± 0.81 ab
Vit. B9	mg 100 g^−1^ DW	0.07 ± 0.01 b	9.38 ± 0.18 a	12.18 ± 0.21 a	7.21 ± 3.66 ab
α-Tocotrienol	µg g^−1^ DW	38.62 ± 0.70 b	75.81 ± 1.39 a	32.86 ± 0.53 b	35.49 ± 2.19 b
Vit. K1	µg g^−1^ DW	6.65 ± 0.13 a	5.59 ± 0.10 ab	5.46 ± 0.10 b	5.99 ± 0.45 ab

Vit. C = ascorbic acid; Vit. B1 = thiamine; Vit. B2 = riboflavin; Vit. B6 = pyridoxine; Vit. B9 = folate (folic acid equivalents); α-Tocotrienol= alpha-tocotrienol, a member of the vitamin E family; Vit. K1 = phylloquinone.

**Table 3:** Average climatic conditions from 1 March to 31 March, including temperature, humidity, and rainfall. The values are presented as mean ± standard deviation for temperature and humidity, while rainfall is shown as total precipitation during the period. (See details in Table S5).

Date	Temperature (°C)	Humidity (%)	Rainfall (mm)
1 March–31 March	14.74 ± 0.47	82.04 ± 1.26	56.4

## Data Availability

Data are contained within the article.

## Supplementary Materials

The following supporting information can be downloaded at: https://www.mdpi.com/article/10.3390/plants15213291/s1, Figure S1: RGB images captured by UAV of four rye genotypes (29 July, 3 September, and 23 October 2025); Figure S2: Multispectral UAV images acquired at three developmental stages of four rye genotypes (29 July, 3 September, and 23 October 2025); Figure S3: Individual variable contributions to the first two dimensions of the multiple factor analysis (MFA) integrating canopy, agronomic, amino acid, and vitamin traits in four rye genotypes. Horizontal bars represent the percentage contribution of the 12 highest-ranking variables to Dimension 1 (61.97% of total inertia; left panel) and Dimension 2 (35.12%; right panel). Colors identify the corresponding data blocks: UAV-derived NDVI (blue), agronomic traits (orange), free amino acids (green), and vitamins (purple). The MFA was performed using genotype-level means (n = 4), comprising three NDVI acquisition dates, leaf area, 18 individual free amino acids, and seven vitamins. Variables were standardized, and blocks were weighted by the inverse square root of their first principal component eigenvalue. Contributions describe the relative importance of individual variables within each dimension and should be interpreted as exploratory rather than inferential; Figure S4: Representative RP-HPLC-DAD chromatogram of FMOC-derivatized free amino acids. Peak identities were assigned by comparison with the amino acid standard mixture analyzed under the same chromatographic conditions. The chromatogram is provided to illustrate the chromatographic separation achieved and the retention-time-based identification of amino acids, including the region corresponding to lysine; Table S1: Analysis of variance for the effects of genotype, sowing date, sampling date, and their interactions on UAV-derived normalized difference vegetation index (NDVI) in rye. Fixed effects were evaluated using a linear mixed-effects model with plot included as a random effect to account for repeated measurements across sampling dates. Denominator degrees of freedom were estimated using Satterthwaite’s approximation. Significant effects are indicated at *p* ≤ 0.05; Table S2: Temporal NDVI measurements and derived canopy descriptors for four rye genotypes sown on 27 March 2025; Table S3: One-way ANOVA summary evaluating the effect of genotype on free amino acid and vitamin composition of rye. Original *p*-values were adjusted for multiple testing across the 25 evaluated compounds (18 free amino acids and seven vitamins) using the Benjamini–Hochberg procedure to control the false discovery rate (FDR) at 5%; Table S4: Exploratory Spearman rank correlations between temporal NDVI descriptors and grain nutritional traits in four rye genotypes; Table S5: Daily climatic parameters recorded during March 2025 at the Maquehue Experimental Field; Table S6: Monthly climatic conditions recorded at the Maquehue Experimental Field during the rye growing season (March 2025–February 2026). Values represent monthly mean air temperature (°C), mean relative humidity (%), and cumulative monthly precipitation (mm); Table S7: Analytical validation parameters for the determination of free amino acids and fat-soluble vitamins by high-performance liquid chromatography with diode-array detection (HPLC-DAD).
