# Supplementary material for: Integrating Canopy Dynamics and Grain Nutritional Profiles Reveals Genotypic Variation in Rye (Secale cereale L.)
Source: Plants (Basel). 2026 Oct 25;15(21):3291. doi: 10.3390/plants15213291 (PMC13765608; doi:10.3390/plants15213291)
Supplement: Supplementary file 1 [file plants-15-03291-s001.zip › plants-4569171-supplementary.pdf]

SUPPLEMENTARY MATERIAL

Table S1. Analysis of variance for the effects of genotype, sowing date, sampling date, and their interactions on UAV-derived normalized difference vegetation index (NDVI) in rye. Fixed effects were evaluated using a linear mixed-effects model with plot included as a random effect to account for repeated measurements across sampling dates. Denominator degrees of freedom were estimated using Satterthwaite's approximation. Significant effects are indicated at $p \leq 0.05$.

Effect	NumDF	DenDF	F value	*p* value
Genotype	3	24.112	33.921	<0.001
Sowing date	1	24.113	2.195	0.151
Sampling date	2	47.250	470.556	<0.001
Genotype × Sowing date	3	24.112	1.789	0.176
Genotype × Sampling date	6	47.240	17.699	<0.001
Sowing date × Sampling date	2	47.250	7.402	0.0016
Genotype × Sowing date × Sampling date	6	47.240	4.078	0.0022

Abbreviations: NumDF, numerator degrees of freedom; DenDF, denominator degrees of freedom; NDVI, normalized difference vegetation index.

Table S2. Temporal NDVI measurements and derived canopy descriptors for four rye genotypes sown on 27 March 2025.

Genotype	NDVI 29 Jul	NDVI 03 Sep	NDVI 23 Oct	Δ Jul–Sep	Δ Sep–Oct	Δ Jul–Oct	Slope (day^{-1})	AUC (NDVI·day)	Normalized AUC
Generator	0.2933	0.2900	0.4525	−0.0033	0.1625	0.1592	0.001851	29.0625	0.3379
Bar Power	0.1050	0.0975	0.3925	−0.0075	0.2950	0.2875	0.003343	15.8950	0.1848
L-INIA	0.0825	0.0675	0.3725	−0.0150	0.3050	0.2900	0.003372	13.7000	0.1593
Lunator	0.2500	0.2475	0.4225	−0.0025	0.1750	0.1725	0.002006	25.7050	0.2989

Table note: NDVI values represent genotype-level means for the 27 March 2025 sowing. Temporal changes (ΔNDVI) were calculated as differences between acquisition dates. Overall temporal slope was calculated over 86 days. AUC was estimated using trapezoidal integration, accounting for the 36-day and 50-day intervals between acquisitions. Normalized AUC was calculated as AUC divided by 86 days. All descriptors were calculated using genotype means and do not represent verified individual plot trajectories.

Table S3. One-way ANOVA summary evaluating the effect of genotype on free amino acid and vitamin composition of rye. Original p-values were adjusted for multiple testing across the 25 evaluated compounds (18 free amino acids and seven vitamins) using the Benjamini–Hochberg procedure to control the false discovery rate (FDR) at 5%. Statistical significance was determined using FDR-adjusted p-values. Significance levels: ns = not significant (adjusted $p > 0.05$); * = adjusted $p \leq 0.05$; ** = adjusted $p \leq 0.01$; *** = adjusted $p \leq 0.001$. Adjusted values were calculated from the reported p-values; for vitamin E, the p-value was estimated from the reported F-statistic and degrees of freedom.

Trait	DF (Genotype, Error)	F-value	p-value	FDR-adjusted p-value (BH)	Significance
L-Arg	3, 8	6.97	0.0127	0.0161	*
L-Gln	3, 8	6.82	0.0135	0.0161	*
L-Ser	3, 8	7.46	0.0105	0.0154	*
L-Asp	3, 8	7.55	0.0102	0.0154	*
L-Glu	3, 8	7.95	0.0088	0.0154	**
L-Thr	3, 8	7.92	0.0088	0.0154	**
L-Gly	3, 8	7.72	0.0095	0.0154	**
L-Ala	3, 8	7.51	0.0103	0.0154	*
L-Pro	3, 8	7.48	0.0104	0.0154	*
L-Met	3, 8	6.97	0.0127	0.0161	*
L-Val	3, 8	7.96	0.0087	0.0154	**
L-Phe	3, 8	3.58	0.0662	0.0690	ns
L-Ile	3, 8	4.91	0.0320	0.0363	*
L-Leu	3, 8	7.96	0.0087	0.0154	**
L-Cys	3, 8	7.90	0.0089	0.0154	**
L-His	3, 8	7.55	0.0101	0.0154	*
L-Tyr	3, 8	7.94	0.0088	0.0154	**
L-Lys	3, 8	0.28	0.8405	0.8405	ns
Vit. C	3, 8	7.60	0.0100	0.0154	**
Vit. B1	3, 8	7.95	0.0088	0.0154	**
Vit. B2	3, 8	6.82	0.0135	0.0161	*
Vit. B6	3, 8	7.94	0.0088	0.0154	**
Vit. B9	3, 8	7.96	0.0087	0.0154	**
Vit. E	3, 8	218.60	<0.0001	<0.0001	***
Vit. K1	3, 8	4.82	0.0334	0.0363	*

Table S4. Exploratory Spearman rank correlations between temporal NDVI descriptors and grain nutritional traits in four rye genotypes.

Nutritional trait	NDVI Jul	NDVI Sep	NDVI Oct	Δ Jul–Sep	Δ Sep–Oct	Slope	AUC
Crude protein	0.40	0.40	0.40	−0.20	−0.40	−0.40	0.40
Arginine	0.20	0.20	0.20	0.40	−0.20	−0.20	0.20
Glutamine	0.40	0.40	0.40	0.00	−0.40	−0.40	0.40
Serine	−0.80	−0.80	−0.80	−0.60	0.80	0.80	−0.80
Aspartate	−0.80	−0.80	−0.80	−0.40	0.80	0.80	−0.80
Glutamate	0.40	0.40	0.40	0.00	−0.40	−0.40	0.40
Threonine	0.80	0.80	0.80	0.60	−0.80	−0.80	0.80
Glycine	−0.40	−0.40	−0.40	−0.20	0.40	0.40	−0.40
Alanine	−0.40	−0.40	−0.40	−0.20	0.40	0.40	−0.40
Proline	−0.80	−0.80	−0.80	−0.60	0.80	0.80	−0.80
Methionine	0.80	0.80	0.80	0.40	−0.80	−0.80	0.80
Valine	0.40	0.40	0.40	0.20	−0.40	−0.40	0.40
Phenylalanine	0.80	0.80	0.80	0.60	−0.80	−0.80	0.80
Isoleucine	−1.00	−1.00	−1.00	−0.80	1.00	1.00	−1.00
Leucine	1.00	1.00	1.00	0.80	−1.00	−1.00	1.00
Cysteine	0.80	0.80	0.80	0.60	−0.80	−0.80	0.80
Histidine	0.80	0.80	0.80	0.60	−0.80	−0.80	0.80
Tyrosine	−1.00	−1.00	−1.00	−0.80	1.00	1.00	−1.00
Lysine	0.40	0.40	0.40	0.20	−0.40	−0.40	0.40
Vitamin C	−0.20	−0.20	−0.20	−0.40	0.20	0.20	−0.20
Vitamin B1	−0.80	−0.80	−0.80	−0.60	0.80	0.80	−0.80
Vitamin B2	0.95	0.95	0.95	0.74	−0.95	−0.95	0.95
Vitamin B6	−0.80	−0.80	−0.80	−0.60	0.80	0.80	−0.80
Vitamin B9	−1.00	−1.00	−1.00	−0.80	1.00	1.00	−1.00
α-Tocotrienol	0.40	0.40	0.40	0.20	−0.40	−0.40	0.40
Vitamin K1	1.00	1.00	1.00	0.80	−1.00	−1.00	1.00

Table note: Values are Spearman's rank correlation coefficients (ϱ) calculated using genotype-level means ($n = 4$). Exact two-sided permutation tests were performed for all associations, and the Benjamini–Hochberg procedure was applied across the complete family of 234 comparisons, including the additional descriptors ΔNDVI July–October and normalized AUC. None of the associations was statistically significant (minimum exact $p = 0.0833$; minimum adjusted $p = 0.4875$). The associations are exploratory and do not establish plot-level relationships or predictive performance. Exact and adjusted p-values should be provided alongside the complete correlation results in the final supplementary dataset.

Table S5. Daily climatic parameters recorded during March 2025 at the Maquehue Experimental Field.

Time UTC-4	Temperature (ºC)	Humidity (%)	Rainfall (mm)
01-03-2025	NA	NA	NA
02-03-2025	NA	NA	NA
03-03-2025	NA	NA	NA
04-03-2025	NA	NA	NA
05-03-2025	NA	NA	NA
06-03-2025	NA	NA	NA
07-03-2025	18.2	90.7	6.5
08-03-2025	16.2	82.4	7.1
09-03-2025	15.3	86.1	0.2
10-03-2025	19.2	79.7	0
11-03-2025	16.9	71.2	0.1
12-03-2025	18.3	86.6	1.7
13-03-2025	16.1	77.6	0.1
14-03-2025	15.2	68.5	0
15-03-2025	16.8	81.5	2
16-03-2025	15.6	93.2	18.8
17-03-2025	12.3	96.3	7.5
18-03-2025	16.6	89.6	0
19-03-2025	13.6	82.6	0
20-03-2025	13.2	80.4	0
21-03-2025	15	80.4	0.1
22-03-2025	15.1	82.3	0
23-03-2025	14.3	77.7	0
24-03-2025	14.2	81.2	0.2
25-03-2025	13.8	80.9	0.1
26-03-2025	13.9	77.5	0.1
27-03-2025	12	88.8	1.3
28-03-2025	13.8	80.4	10.3
29-03-2025	10.6	78.3	0.2
30-03-2025	9	80.8	0
31-03-2025	13.2	76.5	0.1

Table S6. Monthly climatic conditions recorded at the Maquehue Experimental Field during the rye growing season (March 2025–February 2026). Values represent monthly mean air temperature (°C), mean relative humidity (%), and cumulative monthly precipitation (mm).

Month	Temperature (°C)	Relative humidity (%)	Precipitation (mm)
March 2025	14.7	82.5	56.4
April 2025	12.0	87.5	153.7
May 2025	8.8	92.6	122.5
June 2025	6.5	92.0	122.8
July 2025	7.7	91.7	107.0
August 2025	9.9	88.9	107.8
September 2025	10.6	82.7	111.1
October 2025	11.6	80.8	51.4
November 2025	14.8	80.2	26.0
December 2025	16.2	79.3	43.7
January 2026	18.3	73.5	6.6
February 2026	16.6	77.1	42.6

Note: Temperature and relative humidity values represent monthly means, whereas precipitation values correspond to monthly accumulated totals. All meteorological data were recorded at the Maquehue Experimental Field, La Araucanía Region, Chile.

Table S7. Analytical validation parameters for the determination of free amino acids and fat-soluble vitamins by high-performance liquid chromatography with diode-array detection (HPLC-DAD).

Analyte	Linearity (R^2)	LOD	LOQ	Recovery (%)	Precision (CV, %)
Free amino acids					
Arginine	0.9999	0.950	2.879	97.42	2.0
Glutamine	0.9999	0.700	2.121	96.85	2.0
Serine	0.9999	0.550	1.667	95.74	2.0
Aspartic acid	0.9999	0.041	0.124	97.16	2.0
Glutamic acid	0.9999	0.650	1.970	96.38	2.0
Threonine	0.9999	0.800	2.424	94.92	2.0
Glycine	0.9999	0.480	1.455	97.81	2.0
Alanine	0.9999	0.440	1.333	96.53	2.0
Proline	0.9999	0.900	2.727	95.28	2.0
Methionine	0.9999	1.350	4.091	94.67	2.0
Valine	0.9999	1.100	3.333	97.35	2.0
Phenylalanine	0.9999	0.629	1.905	96.12	2.0
Isoleucine	0.9989	1.841	5.578	95.91	2.0
Leucine	0.9999	1.000	3.030	97.68	2.0
Cysteine	0.9993	1.519	4.603	94.46	2.0
Histidine	0.9999	1.150	3.485	96.79	2.0
Tyrosine	0.9971	3.128	9.480	95.37	2.0
Lysine	0.9999	1.300	3.939	97.94	2.0
Fat-soluble vitamins					
α-Tocotrienol	0.9999	0.100	0.303	96.26	3.0
Vitamin K1	0.9999	0.150	0.455	92.88	3.0

Note: R^2, coefficient of determination; LOD, limit of detection; LOQ, limit of quantification; CV, coefficient of variation. LOD and LOQ are expressed in µM for free amino acids and µg mL^{-1} for fat-soluble vitamins.

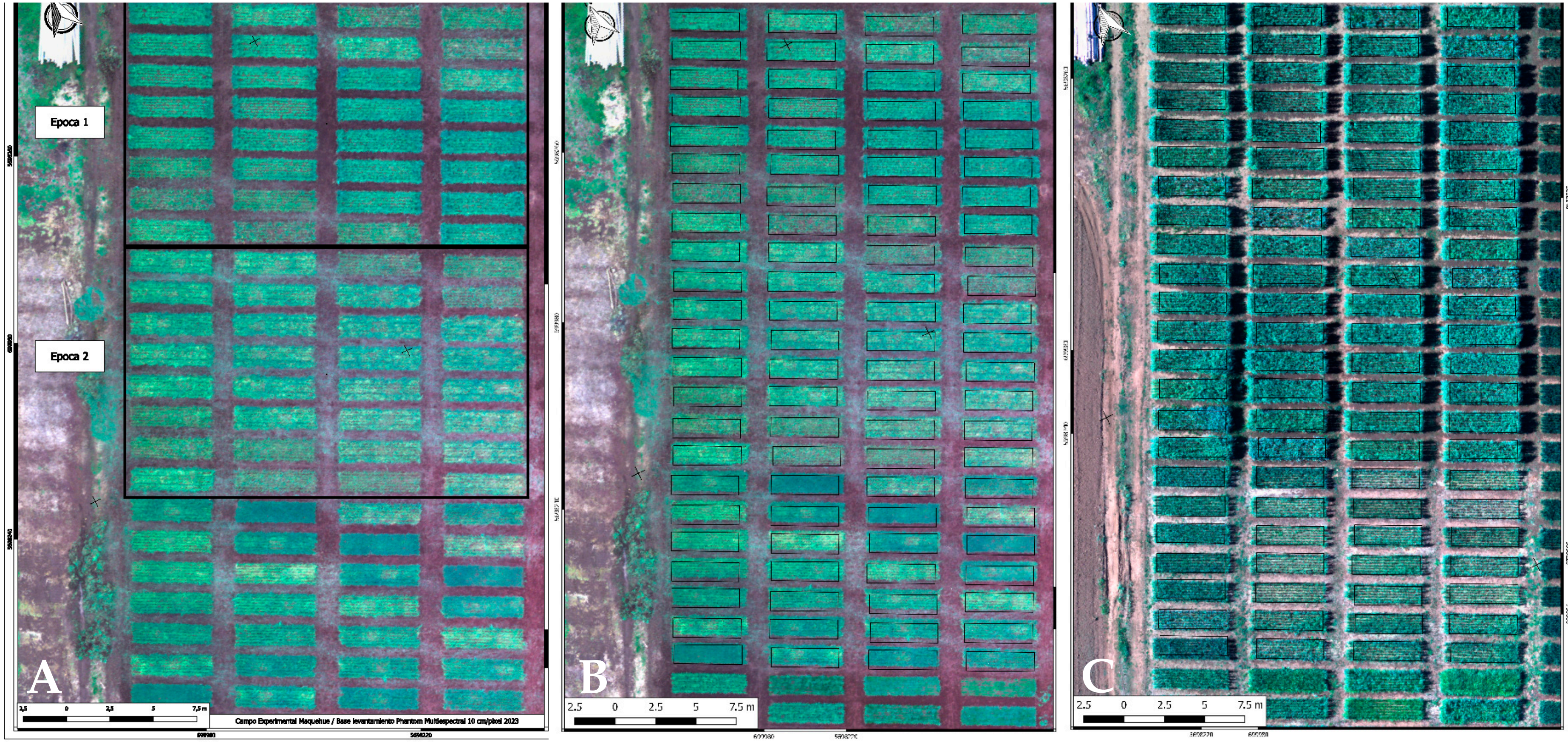

Figure S1. RGB images captured by UAV of four rye genotypes (29 July, 3 September, and 23 October 2025).

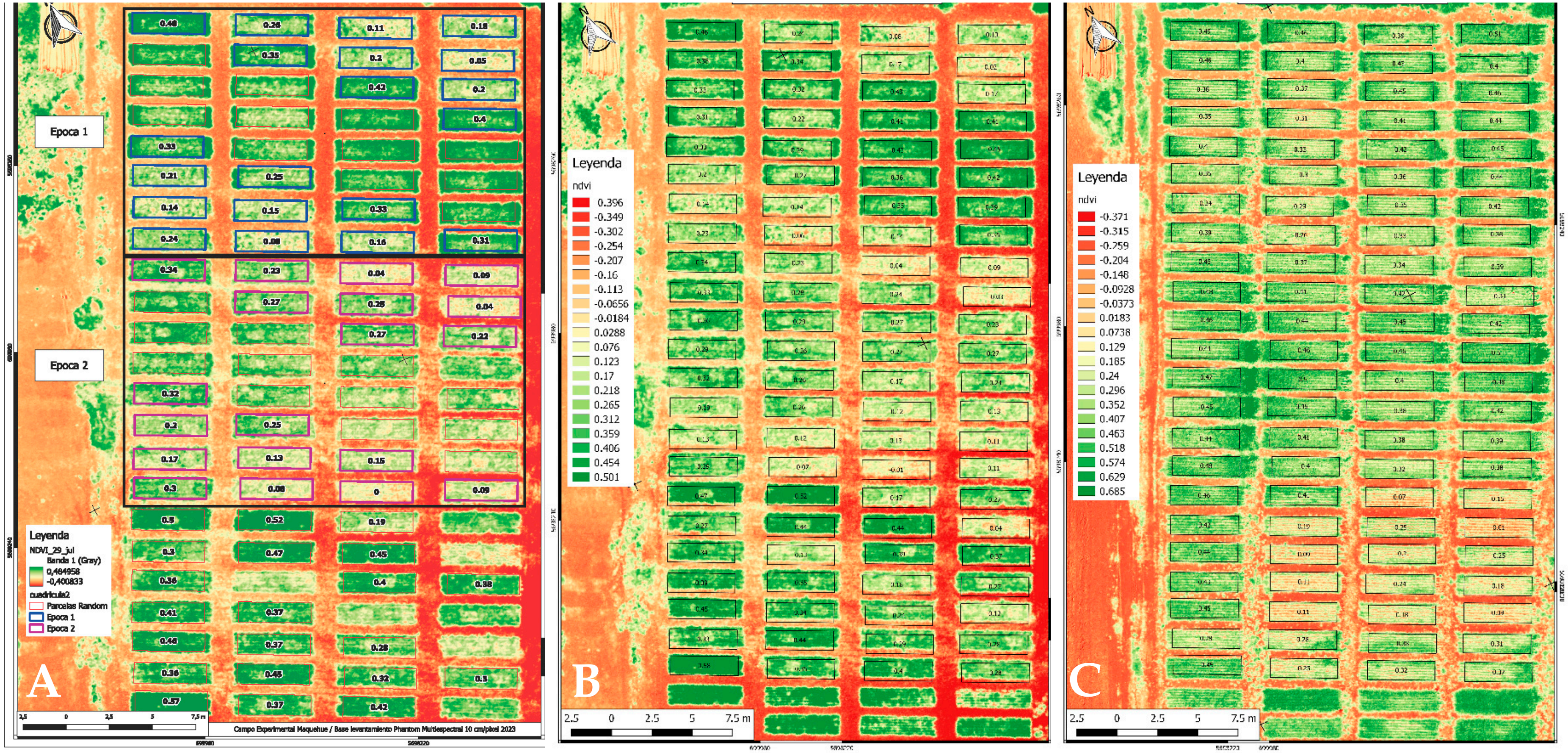

Figure S2. Multispectral UAV images acquired at three developmental stages of four rye genotypes (29 July, 3 September, and 23 October 2025).

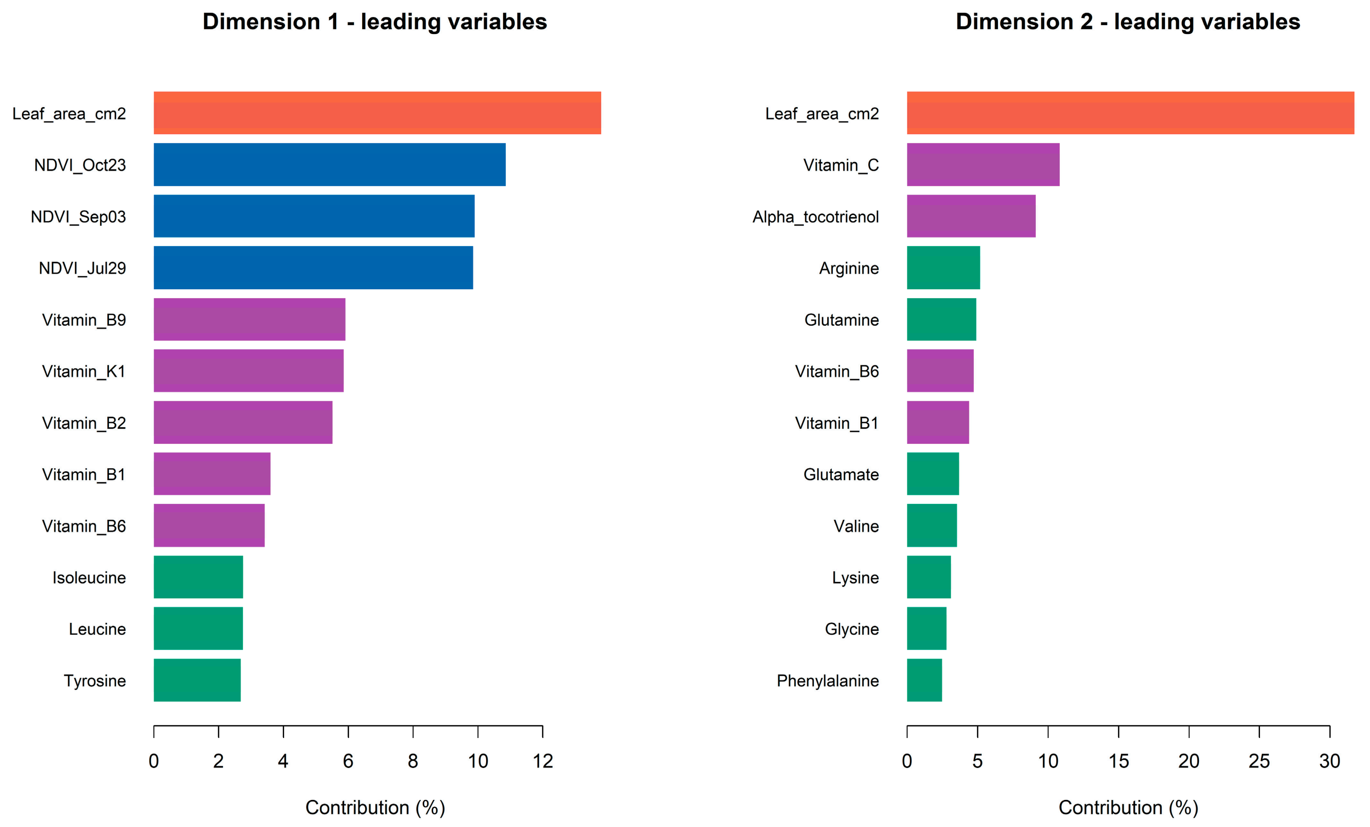

Figure S3. Individual variable contributions to the first two dimensions of the multiple factor analysis (MFA) integrating canopy, agronomic, amino acid, and vitamin traits in four rye genotypes. Horizontal bars represent the percentage contribution of the 12 highest-ranking variables to Dimension 1 (61.97% of total inertia; left panel) and Dimension 2 (35.12%; right panel). Colors identify the corresponding data blocks: UAV-derived NDVI (blue), agronomic traits (orange), free amino acids (green), and vitamins (purple). The MFA was performed using genotype-level means (n = 4), comprising three NDVI acquisition dates, leaf area, 18 individual free amino acids, and seven vitamins. Variables were standardized, and blocks were weighted by the inverse square root of their first principal component eigenvalue. Contributions describe the relative importance of individual variables within each dimension and should be interpreted as exploratory rather than inferential.

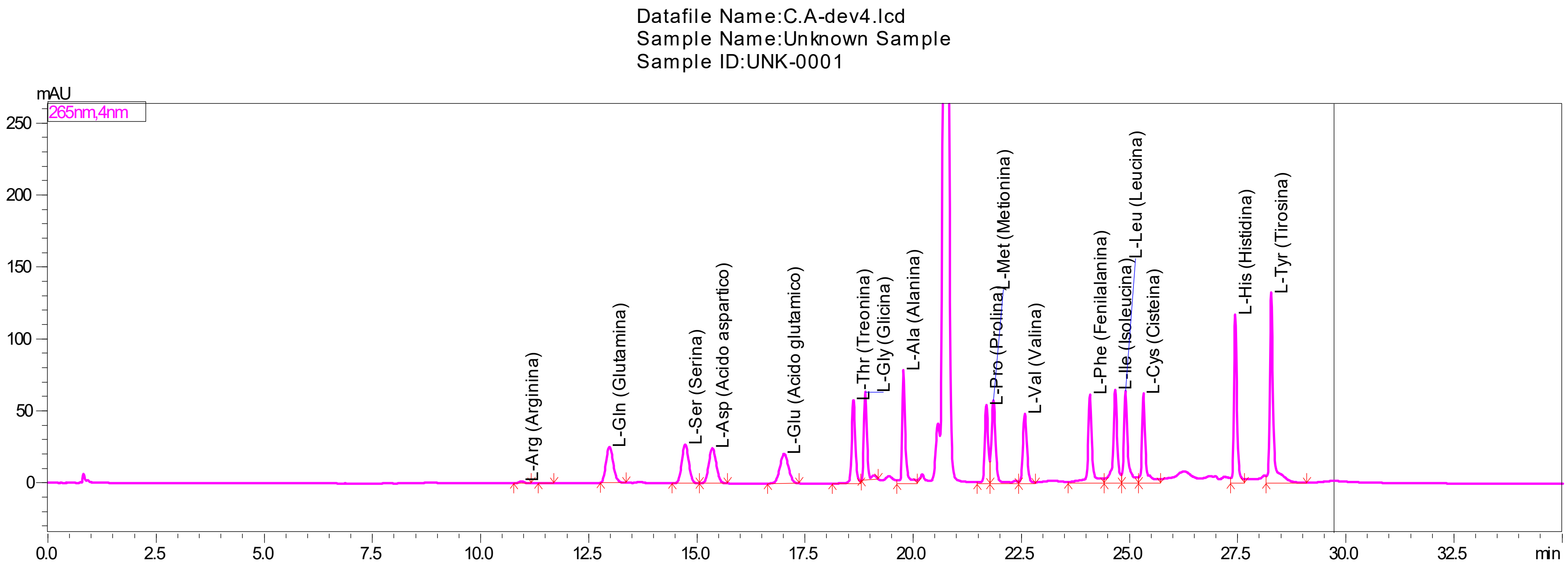

Figure S4. Representative RP-HPLC-DAD chromatogram of FMOC-derivatized free amino acids. Peak identities were assigned by comparison with the amino acid standard mixture analyzed under the same chromatographic conditions. The chromatogram is provided to illustrate the chromatographic separation achieved and the retention-time-based identification of amino acids, including the region corresponding to lysine.
